# 3D deep convolutional neural networks for amino acid environment similarity analysis

**DOI:** 10.1186/s12859-017-1702-0

**Published:** 2017-06-14

**Authors:** Wen Torng, Russ B. Altman

**Affiliations:** 1 Deparment of Bioengineering, Stanford University, Stanford, CA 94305 USA; 2 Department of Genetics, Stanford University, Stanford, CA 94305 USA

**Keywords:** Protein structural analysis, Amino acid similarities, Mutation analysis, Structural bioinformatics, Convolutional neural network, Deep learning

## Abstract

**Background:**

Central to protein biology is the understanding of how structural elements give rise to observed function. The surfeit of protein structural data enables development of computational methods to systematically derive rules governing structural-functional relationships. However, performance of these methods depends critically on the choice of protein structural representation. Most current methods rely on features that are manually selected based on knowledge about protein structures. These are often general-purpose but not optimized for the specific application of interest.

In this paper, we present a general framework that applies 3D convolutional neural network (3DCNN) technology to structure-based protein analysis. The framework automatically extracts task-specific features from the raw atom distribution, driven by supervised labels. As a pilot study, we use our network to analyze local protein microenvironments surrounding the 20 amino acids, and predict the amino acids most compatible with environments within a protein structure. To further validate the power of our method, we construct two amino acid substitution matrices from the prediction statistics and use them to predict effects of mutations in T4 lysozyme structures.

**Results:**

Our deep 3DCNN achieves a two-fold increase in prediction accuracy compared to models that employ conventional hand-engineered features and successfully recapitulates known information about similar and different microenvironments. Models built from our predictions and substitution matrices achieve an 85% accuracy predicting outcomes of the T4 lysozyme mutation variants. Our substitution matrices contain rich information relevant to mutation analysis compared to well-established substitution matrices. Finally, we present a visualization method to inspect the individual contributions of each atom to the classification decisions.

**Conclusions:**

End-to-end trained deep learning networks consistently outperform methods using hand-engineered features, suggesting that the 3DCNN framework is well suited for analysis of protein microenvironments and may be useful for other protein structural analyses.

**Electronic supplementary material:**

The online version of this article (doi:10.1186/s12859-017-1702-0) contains supplementary material, which is available to authorized users.

## Background

Protein sites are microenvironments within a protein structure, distinguished by their structural or functional role. A site can be defined by a three-dimensional location and a local neighborhood around this location in which the structure or function exists. Central to rational protein engineering is the understanding of how the structural arrangement of amino acids creates functional characteristics within protein sites.

Determination of the structural and functional roles of individual amino acids within a protein provides information to help engineer and alter protein functions. Identifying functionally or structurally important amino acids allows focused engineering efforts such as site-directed mutagenesis for altering targeted protein functional properties [1]. Alternatively, this knowledge can help avoid engineering designs that would abolish a desired function. Traditionally, experimental mutation analysis is used to determine the effect of changing individual amino acids. For example, in Alanine scanning, each amino acid in a protein is mutated into Alanine, and the corresponding function or structural effects recorded to identify the amino acids that are critical [2]. This technique is often used in protein-protein interaction hot spot detection for identifying potential interacting residues [3]. However, these experimental approaches are time-consuming and labor-intensive. Furthermore, there is no information about which amino acids would be tolerated at these positions.

The increase in protein structural data provides an opportunity to systematically study the underlying pattern governing such relationships using data-driven approaches. A fundamental aspect of any computational protein analysis is how protein structural information is represented [4, 5]. The performance of machine learning methods often depends more on the choice of data representation than the machine learning algorithm employed. Good representations efficiently capture the most critical information while poor representations create a noisy distribution with no underlying patterns.

Most methods rely on features that have been manually selected based on understanding sources of protein stability and chemical composition. For example, property-based representations describe physicochemical properties associated with local protein environments in protein structures using biochemical features of different level of details [6–9]. Zvelebil et al. have shown that properties including residue type, mobility, polarity, and sequence conservation are useful to characterize the neighborhood of catalytic residues [9]. The FEATURE program [6], developed by our group, represents protein microenvironments using 80 physicochemical properties. FEATURE divides the local environment around a point of interest into six concentric shells, each of 1.25 Å in thickness, and evaluates the 80 physicochemical properties within each shell. The properties range from low-level features such as atom type or the presence of residues to higher-level features such as secondary structure, hydrophobicity and solvent accessibility. We have applied the FEATURE program to different important biological problems, including the identification of functional sites [10], characterization of protein pockets [11], and prediction of interactions between protein pockets and small molecules [12], with success.

However, designing hand-engineered features is labor-intensive, time-consuming, and not optimal for some tasks. For example, although robust and useful, the FEATURE program has several limitations [6, 11, 13]. To begin with, each biological question depends on different sets of protein properties and no single set encodes all the critical information for each application. Second, FEATURE employs 80 physiochemical features with different level of details; some attributes have discrete values, while others are real valued. The high dimensionality together with the inhomogeneity among the attributes can be challenging for machine learning algorithms [14]. Finally, FEATURE use concentric shells to describe local microenvironments. The statistics of biochemical features within each shell are collected but information about the relative position within each shell is lost. The system is therefore rotational invariant but can fail in cases where orientation specific interactions are crucial.

The surfeit of protein structures [15] and the recent success of deep learning algorithms provide an opportunity to develop tools for automatically extracting task specific representations of protein structures. Deep learning networks have achieved great success in computer vision and natural language processing community [16–19], and have been used in small molecule representation [20, 21], transcription factor binding prediction [22], prediction of chromatin effects of sequence alterations [23], and prediction of patient outcome from electronic health records [24]. The power of deep learning lies in its ability to extract useful features from raw data form [16]. Deep convolutional neural networks (CNN) [17, 25] comprise a subclass of deep learning networks. Local filters in CNNs scan through the input space and search for recurring local patterns that are useful for classification performance. By stacking multiple CNN layers, deep CNNs hierarchically compose simple local spatial features into complex features. Biochemical interactions occur locally, and can be aggregated over space to form complicated and abstract interactions. The success of CNNs at extracting features from 2D images suggests that the convolution concept can be extended to 3D and applied to proteins represented as 3D “images”. In fact, Wallach et al. [26] applied 3D convolutional neural networks to protein-small molecule bioactivity predictions and showed that performances of deep learning framework surpass conventional docking algorithms.

In this paper, we develop a general framework that applies 3D convolutional neural networks for protein structural analysis. The strength of our method lies in its ability to automatically extract task-specific features, driven by supervised labels that define the classification goal. Importantly, unlike conventional engineered biochemical descriptors, our 3DCNN requires neither prior knowledge nor assumptions about the features critical to the problem. Protein microenvironments are represented as four atom “channels” (analogous to red, green, blue channels in images) in a 20 Å box around a central location within a protein microenvironment. The algorithm is not dependent on pre-specified features and can discover arbitrary features that are most useful for solving the problem of interest. To demonstrate the utility of our framework, we applied the system to characterize microenvironments of the 20 amino acids. Specifically, we present the following:

1. To study how the 20 amino acids interact with their neighboring microenvironment, we train our network to predict the amino acids most compatible with a specific location within a protein structure. We perform head-to-head comparisons of prediction performance between our 3DCNN and models using the FEATURE descriptors and show that out 3DCNN achieved superior performances over models using conventional features.
2. We demonstrate that the features captured by our network are useful for protein engineering applications. We apply results of our network to predicting effects of mutations in T4 lysozyme structures. We evaluate the extent to which an amino acid “fits” its surrounding protein environment and show that mutations that disrupt strong amino acid preferences are more likely to be deleterious. The prediction statistics over millions of training and test examples provide information about the propensity of each amino acid to be substituted for another. We therefore construct two substitution matrices from the prediction statistics and combine information from the class predictions and the substitution matrices to predict effects of mutation in T4 lysozyme structures.
3. We present a new visualization technique, “atom importance map”, to inspect individual contribution of each atom within the input example to the final decision. The importance map helps us intuitively visualize the features our network has captured.
  Our 3DCNN achieves a two-fold increase in microenvironments prediction accuracies compared to models that employ conventional structure-based hand-engineered biochemical features. Hierarchical clustering of our amino acid prediction statistics confirms that our network successfully recapitulates hierarchical similarities and differences among the 20 amino acid microenvironments. When used to predict effects of mutations in T4 lysozyme structures, our models demonstrate strong ability to predict outcomes of the mutation variants, with 85% accuracy to separate the destabilizing mutations from the neutral ones. We show that substitution matrices built from our prediction statistics encode rich information relevant to mutation analysis. When no structural information is provided, models built from our matrices on average outperform the ones built from BLOSUM62 [27], PAM250 [28] and WAC [29] by 25.4%. Furthermore, given the wild type structure, our network predictions enable the BLOSUM62, PAM250 and WAC models to achieve an average 35.8% increase in prediction accuracies. Finally, the atom input importance visualization confirms that our network recognizes meaningful biochemical interactions between amino acids.

## Methods

### Datasets

#### T4-lysozyme free, protein-family-based training and test protein structure sets

For the 20 amino acid microenvironment classification problem, we construct our dataset based on the SCOP [30] and ASTRAL [31] classification framework (version 1.75.) To avoid prediction biases derived from similar proteins within the same protein families, we ensure that no structure in the training set belongs to the same protein family as any structure in the test set. Specifically, we first retrieved representative SCOP domains from the ASTRAL database. We excluded multi-chain domains, and identified protein families of the representative domains using the SCOP classification framework, resulting in 3890 protein families. We randomly selected 5 % of the identified protein families (194 protein families) from the 3890 protein families to form the test family set—with the remaining 3696 protein families forming the training family set. Member domains of a given protein family were either entirely assigned to training set or entirely assigned to test set. In addition, we removed PDB-IDs present in both the training and test sets to ensure there was no test chain in a family that was used in training. To enforce strict sequence level similarity criteria between our training and test set, we used CD-HIT-2D [32] to identify any test chain that has a sequence similarity above 40% to any chain in the training structure set, and removed the identified structures from the test set.

Furthermore, to obtain fair evaluation of our downstream application that characterizes T4 lysozyme mutant structures, we removed T4 lysozyme structures from both datasets. Specifically, PDB-IDs of the wild-type and mutant T4 lysozyme structures were first obtained from the Uniprot [33] database. We then excluded structures containing domains in the same family as any wild type or mutant T4 lysozyme structure from both the training and test datasets. We obtained the final selected protein structures from the PDB as of date Oct 19 2016.

#### Input Featurization and processing

To facilitate comparison between deep learning and conventional algorithms built with hand-engineered biochemical features, we created two datasets from the same train and test protein structure sets described in T4-lysozyme Free, Protein-Family-Based Training and Test Protein Structure Sets section.

##### (A) Atom-Channel Dataset

ᅟ

#### Local box extraction and labeling

**Fig. 1:**
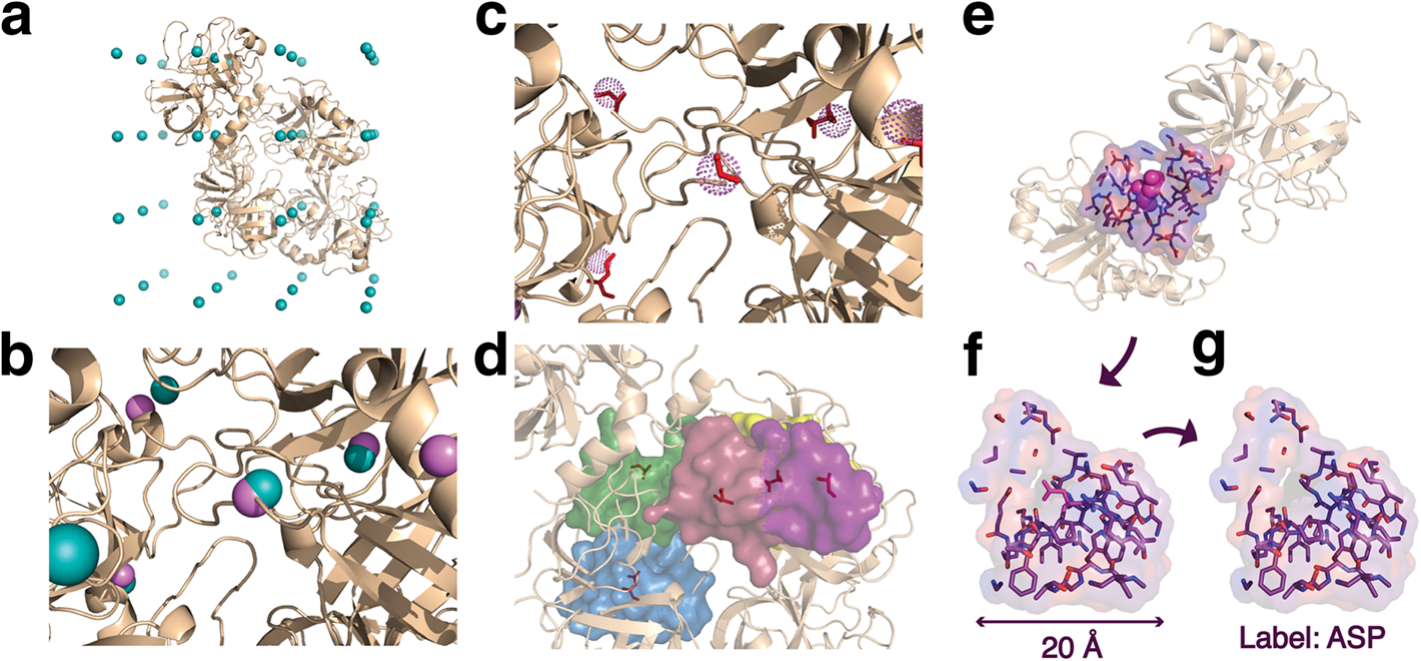
Local box sampling and extraction. **a** For each structure in the training and test structure sets, we placed a 3D grid with 10 Å spacing to sample positions in the protein for local box extraction. The teal spheres represent the sampled grid positions. (For illustration purpose, a grid size of 25 Å instead of 10 Å is shown here). **b** For each sampled position, the nearest atom (*pink sphere*) to the sampled position (*teal sphere*) is first identified. **c** The amino acid which this atom belongs to is then assigned as the central amino acid. The selected amino acids are highlighted in red and the atoms are shown as dotted spheres. **d** A local box of 20 Å is then defined around the central amino acid, centering on the Cβ. For each amino acid microenvironment, a local box is extracted around the amino acid using the following procedure: **e** Backbone atoms of the center amino acid is first used to calculate the orthogonal axes for box extraction. **f** A 20 Å box is extracted around the Cβ atom of the center amino acid using the defined orientation. **g** Side-chain atoms of the center amino acid are removed. The extracted box is then labeled with the removed amino acid side-chain type

For each structure in the training and test structure sets, we placed a 3D grid with 10 Å spacing to sample positions in the protein for local box extraction. Specifically, we first identify the minimum Cartesian x, y and z coordinates of the structure, and define the (x_min_, y_min_, z_min_) position as the origin of our 3D grid. We then construct a 3D grid with 10 Å spacing that covers the whole structure (Fig. 1a.) For each sampled position, a local box is extracted using the following procedure: The nearest atom to the sampled position is first identified (Fig. 1b) and the amino acid which this atom belongs to is assigned as the central amino acid (Fig. 1c). To achieve consistent orientation, each box is aligned within the box in a standard manner using the backbone geometry of the center amino acid (Fig. 1d). Specifically, each box is oriented such that the plane formed by the N-CA and the C-CA bonds forms the x-y plane and the orthogonal orientation with which the CA- Cβ bond has a positive dot product serves as the positive z-axis (Fig. 1e). A 20 Å box is then extracted around the Cβ atom of the central amino acid using the defined orientation (Fig. 1f). We chose the Cβ atom of each amino acid as center to maximize the observable effects of the side chain while still maintaining a comparable site across all 20 amino acids. The Cβ atom position of Glycine was estimated based on the average position of the superimposed Cβ atoms from all other amino acids. Side-chain atoms of the center amino acid are removed. The extracted box is then labeled with the removed amino acid side-chain type (Fig. 1g).

#### Local box Featurization

**Fig. 2:**
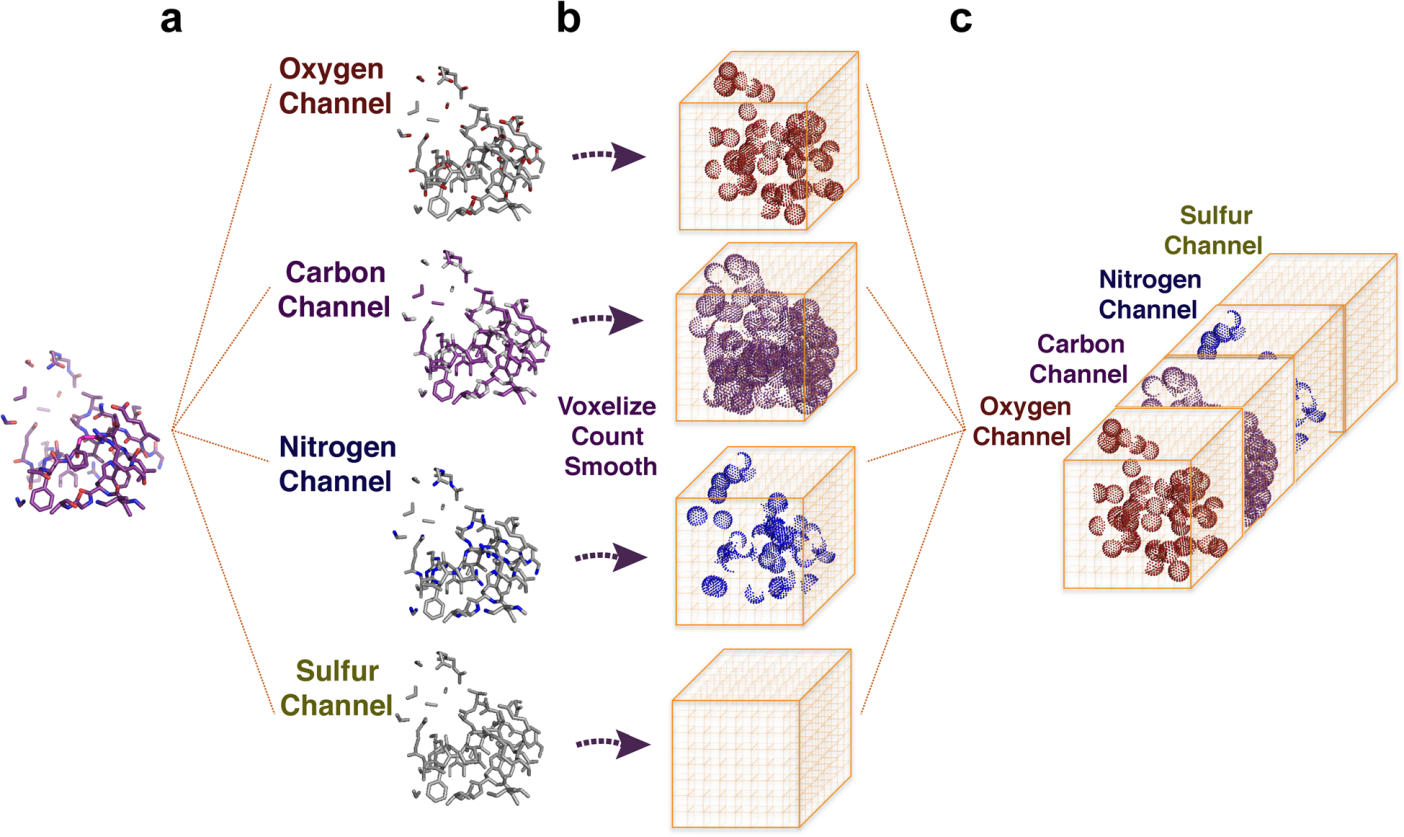
Local box featurization. **a** Local structure in each 20 Å box is first decomposed into Oxygen, Carbon, Nitrogen, and Sulfur channels. **b** Each atom type channel structure is divided into 3D 1-Å voxels, within which the presence of atom of the corresponding atom type is recorded. Within each channel, Gaussian filters are applied to the discrete counts to approximate the atom connectivity and electron delocalization. **c** The resulting numerical 3D matrices of each atom type channel are then stacked together as different input channels, resulting in a (4, 20, 20, 20) 4D–tensor, which will serve as an input example to our 3DCNN

Each local 20 Å box is further divided into 1-Å 3D voxels, within which the presence of carbon, oxygen, sulfur, and nitrogen atoms are recorded in a corresponding atom type channel (Fig. 2.) Although including the hydrogen atoms would provide more information, we did not include them because their positions are almost always deterministically set by the position of the other heavy atoms, and so they are implicitly represented in our networks (and many other computational representations). We believe that our model is able to infer the impact of these implicit hydrogens. The 1-Å voxel size ensures that each voxel can only accommodate a single atom, which could allow our network to achieve better spatial resolution. Given an atom within a voxel, one of the four atom channel types will have a value of 1 in the corresponding voxel position, and the other three channels will have the value 0.

We then apply Gaussian filters to the discrete counts to approximate atom connectivity and electron delocalization. Standard deviation of the Gaussian filters is calibrated to the average Van der Waals radii of the four atom types. The local box extraction and featurization steps are performed on both the training and test protein structure sets to form the training and test dataset.

#### Dataset balancing

Different amino acids have strikingly different frequencies of occurrence within natural proteins. To ensure useful features can be extracted from all the 20 amino acid microenvironment types, we construct balanced training and test datasets by applying the following procedure to the training and test dataset: (1) The least abundant amino acid microenvironment in the original dataset is first identified. (2) All examples of the identified amino acid microenvironment type are included in the balanced dataset. (3) The number of examples for the least abundant amino acid microenvironment is used to randomly sample an equal amount of examples from all the other 19 amino acid microenvironment types. Validation examples are randomly drawn from the balanced training set using a 1:19 ratio. This ensures an approximately equal number of examples from all the 20 amino acid microenvironment types for the balanced training, validation and test datasets.

#### Data normalization

Prior to being fed into the deep learning network, input examples are zero-mean normalized. Specifically, mean values of each channel at each position across the training dataset are calculated and subtracted from the training, validation, and test examples.

##### (B) FEATURE Dataset

ᅟ

#### FEATURE microenvironments

**Fig. 3:**
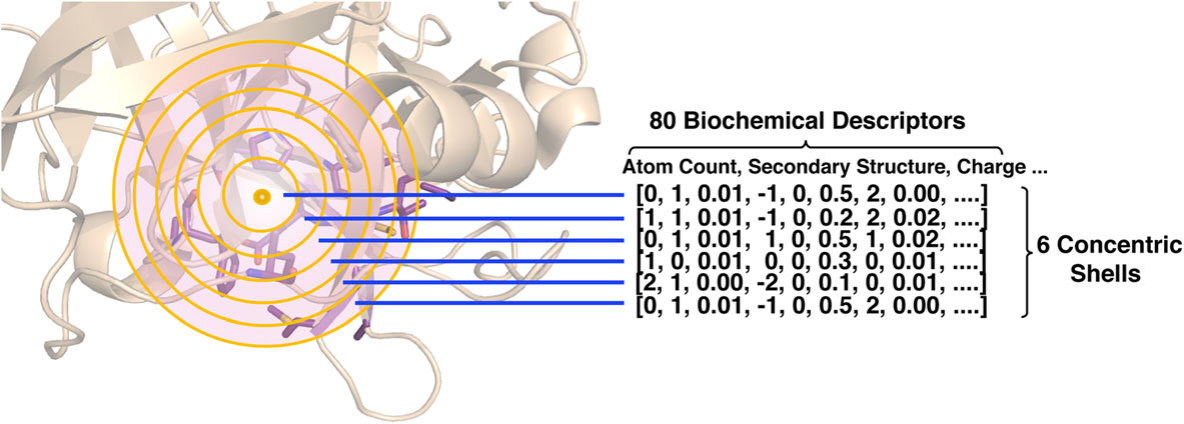
The FEATURE program. FEATURE captures the physicochemical information around a point of interest in protein structure by segmenting the local environment into six concentric shells, each of 1.25 Å in thickness. Within each shell, FEATURE evaluates 80 physicochemical properties including atom type, residue class, hydrophobicity, and secondary structure. This enables conversion of a local structural environment into a numeric vector of length 480

FEATURE, a software program previously developed in our lab, is used as a baseline method to demonstrate the performance of conventional hand-engineered structure-based features [6]. The FEATURE program captures the physicochemical information around a point of interest in protein structure by segmenting the local environment into six concentric shells, each of 1.25 Å in thickness (Fig. 3). Within each shell, FEATURE evaluates 80 physicochemical properties including atom type, residue class, hydrophobicity, and secondary structure (See Table 1 for a full list of the properties). This enables conversion of a local structural environment into a numeric vector of length 480.

**Table 1:** Full list of the 80 biochemical properties used in the FEATURE program

1	ATOM_TYPE_IS_C	41	RESIDUE_NAME_IS_GLU
2	ATOM_TYPE_IS_CT	42	RESIDUE_NAME_IS_GLY
3	ATOM_TYPE_IS_CA	43	RESIDUE_NAME_IS_HIS
4	ATOM_TYPE_IS_N	44	RESIDUE_NAME_IS_ILE
5	ATOM_TYPE_IS_N2	45	RESIDUE_NAME_IS_LEU
6	ATOM_TYPE_IS_N3	46	RESIDUE_NAME_IS_LYS
7	ATOM_TYPE_IS_NA	47	RESIDUE_NAME_IS_MET
8	ATOM_TYPE_IS_O	48	RESIDUE_NAME_IS_PHE
9	ATOM_TYPE_IS_O2	49	RESIDUE_NAME_IS_PRO
10	ATOM_TYPE_IS_OH	50	RESIDUE_NAME_IS_SER
11	ATOM_TYPE_IS_S	51	RESIDUE_NAME_IS_THR
12	ATOM_TYPE_IS_SH	52	RESIDUE_NAME_IS_TRP
13	ATOM_TYPE_IS_OTHER	53	RESIDUE_NAME_IS_TYR
14	PARTIAL_CHARGE	54	RESIDUE_NAME_IS_VAL
15	ELEMENT_IS_ANY	55	RESIDUE_NAME_IS_HOH
16	ELEMENT_IS_C	56	RESIDUE_NAME_IS_OTHER
17	ELEMENT_IS_N	57	RESIDUE_CLASS1_IS_HYDROPHOBIC
18	ELEMENT_IS_O	58	RESIDUE_CLASS1_IS_CHARGED
19	ELEMENT_IS_S	59	RESIDUE_CLASS1_IS_POLAR
20	ELEMENT_IS_OTHER	60	RESIDUE_CLASS1_IS_UNKNOWN
21	HYDROXYL	61	RESIDUE_CLASS2_IS_NONPOLAR
22	AMIDE	62	RESIDUE_CLASS2_IS_POLAR
23	AMINE	63	RESIDUE_CLASS2_IS_BASIC
24	CARBONYL	64	RESIDUE_CLASS2_IS_ACIDIC
25	RING_SYSTEM	65	RESIDUE_CLASS2_IS_UNKNOWN
26	PEPTIDE	66	SECONDARY_STRUCTURE1_IS_3HELIX
27	VDW_VOLUME	67	SECONDARY_STRUCTURE1_IS_4HELIX
28	CHARGE	68	SECONDARY_STRUCTURE1_IS_5HELIX
29	NEG_CHARGE	69	SECONDARY_STRUCTURE1_IS_BRIDGE
30	POS_CHARGE	70	SECONDARY_STRUCTURE1_IS_STRAND
31	CHARGE_WITH_HIS	71	SECONDARY_STRUCTURE1_IS_TURN
32	HYDROPHOBICITY	72	SECONDARY_STRUCTURE1_IS_BEND
33	MOBILITY	73	SECONDARY_STRUCTURE1_IS_COIL
34	SOLVENT_ACCESSIBILITY	74	SECONDARY_STRUCTURE1_IS_HET
35	RESIDUE_NAME_IS_ALA	75	SECONDARY_STRUCTURE1_IS_UNKNOWN
36	RESIDUE_NAME_IS_ARG	76	SECONDARY_STRUCTURE2_IS_HELIX
37	RESIDUE_NAME_IS_ASN	77	SECONDARY_STRUCTURE2_IS_BETA
38	RESIDUE_NAME_IS_ASP	78	SECONDARY_STRUCTURE2_IS_COIL
39	RESIDUE_NAME_IS_CYS	79	SECONDARY_STRUCTURE2_IS_HET
40	RESIDUE_NAME_IS_GLN	80	SECONDARY_STRUCTURE2_IS_UNKNOWN

Description of each property can be found in [63]

#### Dataset construction

Following a similar sampling procedure described in (A) Atom-Channel Dataset section, we placed a 3D grid with 10 Å spacing to sample positions for featurization in each structure in the training and test structure sets (Fig. 1a), where the 3D grid is constructed using the same procedure as in (A) Atom-Channel Dataset section. For each sampled position within a structure, the center residue is determined by identifying the nearest residue (Fig. 1b and c). A modified structure with the center residue removed from the original structure is subsequently generated. The FEATURE software is then applied to the modified structure, using the Cβ atom position of the central residue, and generates a feature vector of length 480 to characterize the microenvironment. The generated training and test datasets are similarly balanced and zero-mean normalized, as described in (A) Atom-Channel Dataset section. Validation examples were randomly drawn from the balanced training set using a 1:19 ratio.

### Network architecture

**Fig. 4:**
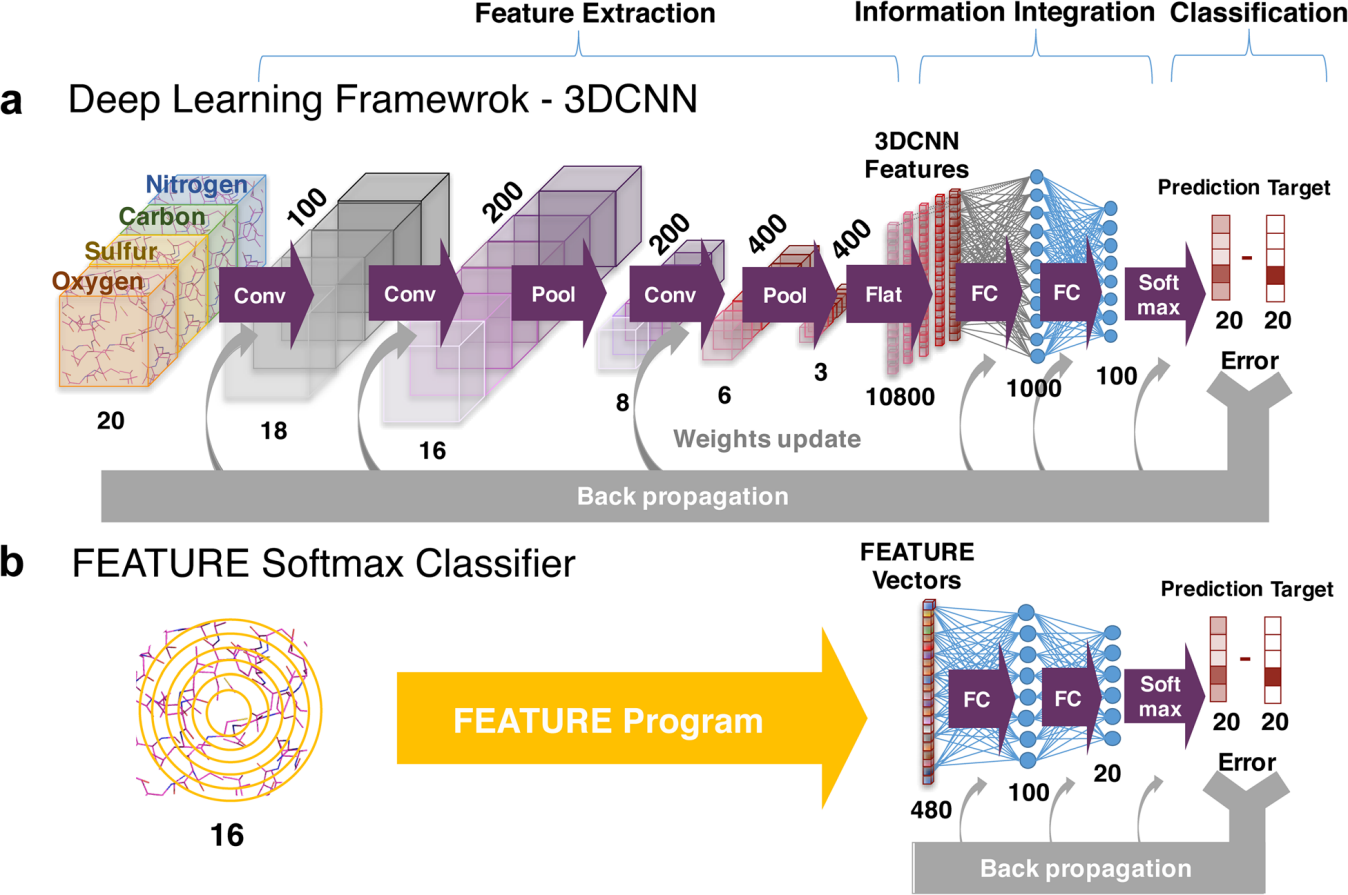
Schematic diagram of the Deep 3D Convolutional Neural Network and FEATURE-Softmax Classifier models. **a** Deep 3D Convolutional Neural Network. The feature extraction stage includes 3D convolutional and max-pooling layers. 3D filters in the 3D convolutional layers search for recurrent spatial patterns that best capture the local biochemical features to separate the 20 amino acid microenvironments. Max Pooling layers perform down-sampling to the input to increase translational invariances of the network. By following the 3DCNN and 3D Max-Pooling layers with fully connected layers, the pooled filter responses of all filters across all positions in the protein box can be integrated. The integrated information is then fed to the Softmax classifier layer to calculate class probabilities and to make the final predictions. Prediction error drives parameter updates of the trainable parameters in the classifier, fully connected layers, and convolutional filters to learn the best feature for the optimal performances. **b** The FEATURE Softmax Classifier. The FEATURE Softmax model begins with an input layer, which takes in FEATURE vectors, followed by two fully-connected layers, and ends with a Softmax classifier layer. In this case, the input layer is equivalent to the feature extraction stage. In contrast to 3DCNN, the prediction error only drives parameter learning of the fully connected layers and classifier. The input feature is fixed during the whole training process

To perform head-to-head comparisons between end-to-end trained deep learning framework that takes in raw input representations and machine learning models that are built on top of conventional hand-engineered features, we design the following two models: (A) Deep 3D Convolutional Neural Network (B) FEATURE Softmax Classifier. Both models comprise three component modules: (1) Feature Extraction Stage (2) Information Integration Stage (3) Classification Stage, as shown in Fig. 4. To evaluate the advantages of using a Deep Convolutional Architecture versus a simple flat neural network, we also built a third model (C) Multi-Layer Perceptron with 2 hidden layers.

#### (A) Deep 3D Convolutional neural network

Our deep 3D convolutional neural network is composed of the following modules: (1) 3D Convolutional Layer (2) 3D Max Pooling Layer [34] (3) Fully Connected Layer (4) Softmax Classifier [35]. In brief, our network begins with three sequential alternating 3D convolutional layers and 3D max pooling layers, which extract 3D biochemical features at different spatial scales, followed by two fully-connected layers which integrate information from the pooled response across the whole input box, and ends with a Softmax classifier layer, which calculates class scores and class probability of each of the 20 amino acid classes. Schematic diagram of the network architecture is shown in Fig. 4. The operation and function of each module are briefly described below. All modules in the network were implemented in Theano [36].

- **3D Convolutional Layer**
  The 3D Convolution layer consists of a set of learnable 3D filters, each of which has small local receptive field that extends across all input channels. During the forward pass, each filter moves across the width, height and depth of the input space with a fixed stride, convolves with its local receptive field at each position and generate filter responses. The rectified linear (ReLU) [37] activation function consecutively performs a non-linear transformation on the filter responses to generate the activation values. More formally, the activation value ${a}_{i, j, k}^L$ at output position (i,j,k) of the L^th^ filter when convolving with the input X can be calculated by Eqs. (1) and (2).

1 $$ {a}_{i, j, k}^L=\mathrm{ReLU}\left[{\sum}_{m= i}^{i+\left( F-1\right)}{\sum}_{n= j}^{j+\left( F-1\right)}{\sum}_{d= k}^{k+\left( F-1\right)}{\sum}_{c=0}^{C-1}{W}_{c, m, n, d}^L{X}_{c, m, n, d}+{b}^L\right] $$

2 $$ \mathrm{ReLU}=\left\{\begin{array}{c}\hfill x,\kern0.5em  if\  x\ge 0\hfill \\ {}\hfill 0,\kern0.5em  if\  x<0\hfill \end{array}\right. $$

Where F is the filter size, assuming the filter has equal width, height and depth, C is the number of input channels, W is a weight matrix with size (C,F,F,F), X is the input, i, j, k are the indices of the output position, and m, n, d are the indices of the input position.

Our 3D Convolution module takes in a 5D–tensor of shape [batch size, number of input channels, input width, input height, input depth], convolves the 5D–tensor with 3D filters of shape [number of input channels, filter width, filter height, filter depth] with stride 1, and outputs a 5D- tensor of shape [batch size, number of 3D filters, (input width- filter width) +1, (input height- filter height) +1, (input depth - filter depth) +1]. During the training process, the weights of each of the 3D convolutional filters are optimized to detect local spatial patterns that best capture the local biochemical features to separate the 20 amino acid microenvironments. After the training process, filters in the 3D convolution layer will be activated when the desired features are present at some spatial position in the input.

- **3D Max Pooling Layer**

The 3D max pooling module takes in an input 5D–tensor of shape [batch size, number of input channels, input width, input height, input depth], performs down-sampling of the input tensor with stride of 2, and output a 5D- tensor of shape [batch size, number of input channels, input width/2, input height/2, input depth/2]. For each channel, the max pooling operation identifies the maximum response value for each 2*2*2 subregion and reduce the 2*2*2 cube region into a single 1*1*1 cube with the representative maximum value. The operation can be described by Eq. (3).

3 $$ {\mathrm{MP}}_{\mathrm{c},\mathrm{l},\mathrm{m},\mathrm{n}}= \max \left(\left\{{\mathrm{X}}_{\mathrm{c},\mathrm{i},\mathrm{j},\mathrm{k}},{\mathrm{X}}_{\mathrm{c},\mathrm{i}+1,\mathrm{j},\mathrm{k}},{\mathrm{X}}_{\mathrm{c},\mathrm{i},\mathrm{j}+1,\mathrm{k}},{\mathrm{X}}_{\mathrm{c},\mathrm{i},\mathrm{j},\mathrm{k}+1},{\mathrm{X}}_{\mathrm{c},\mathrm{i}+1,\mathrm{j}+1,\mathrm{k}},{\mathrm{X}}_{\mathrm{c},\mathrm{i},\mathrm{j}+1,\mathrm{k}+1},{\mathrm{X}}_{\mathrm{c},\mathrm{i}+1,\mathrm{j},\mathrm{k}+1},{\mathrm{X}}_{\mathrm{c},\mathrm{i}+1,\mathrm{j}+1,\mathrm{k}+1}\right\}\right) $$

Where $$ \left\{\begin{array}{c} i={l}^{\ast }2\\ {} j={m}^{\ast }2\\ {} k={n}^{\ast }2.\end{array}\right. $$

*MP denotes the output of the Max-Pooling operation of X

*l, m, n are the indices of the output position, c denotes the input channel, and i, j, k are the indices of the input position

- **Fully Connected Layer and the Softmax Classifier**

The fully-connected layer integrates information of neurons across all positions within a layer using a weight matrix that connect all neurons in the layer to all neurons in the subsequent layer. A ReLU function follows to perform a non-linear transformation. The operation is described by Eq. (4). By following the 3DCNN and 3D Max-Pooling layers with fully connected layers, the pooled filter responses of all filters across all positions in the protein box can be integrated. The integrated information is then fed to the Softmax classifier layer to calculate class probabilities and to make the final predictions.

4 $$ {h}_n=\mathrm{ReLU}\left(\sum_{m=0}^{M-1}{W}_{m, n}{X}_m+{b}_n\ \right) $$

Where *h*_*n*_ denotes the activation value of the n^th^ neuron in the output layer, M denotes the number of neurons in the input layer, N denotes the number of neurons in the output layer, and W is a weight matrix with size [M, N].

#### (B) FEATURE Softmax classifier

The FEATURE Softmax Classifier model comprises the same three feature extraction, information integration and classification stages. The model begins with an input layer, which takes in FEATURE vectors generated in (B) FEATURE Dataset section. In this case, the input layer is equivalent to the feature extraction stage since the biochemical features are extracted from the protein structures by the FEATURE program prior to being fed into the model. The input layer is then followed by two fully-connected layers, which integrate information from the input features. Finally, the model ends with a Softmax classifier layer, which performs the classification.

#### (C) Multi-Layer Perceptron

Our Multi-Layer Perceptron model takes in the same local boxes input as the 3DCNN model, flattens the 5D–tensor of shape (batch size, number of input channels, input width, input height, input depth) into a 2D matrix of shape (batch size, number of input channels* input width*input height*input depth), and has just two fully-connected layers which integrate information across the whole input box, ending with a Softmax classifier layer.

We trained our 3DCNN, MLP, and the FEATURE Softmax Classifier using stochastic gradient descent [38] with the back-propagation algorithm [39]. Gradients were computed by the automatic differentiation function implemented in Theano. A batch size of 20 examples was used. To avoid over-fitting, we used L2 regularization for all the models, and employed dropout [40] (*p* = 0.3) when training the 3DCNN, FEATURE Softmax Classifier and MLP. We tested different L2 regularization constants and dropout rates. We selected the appropriate L2 regularization constant and dropout rate based on validation set performance; we did not attempt to optimize the other meta-parameters. We trained the 3DCNN network for 6 days for 9 epochs using GPUs on the Stanford Xstream cluster. The MLP model was trained for 20 epochs using GPUs on the Stanford Xstream cluster until convergence. The FEATURE Softmax classifier took 3 days on the Stanford Sherlock cluster to reach convergence. The Stanford XStream GPU cluster is made of 65 compute nodes for a total of 520 Nvidia K80 GPU cards (or 1040 logical graphical processing units). The Stanford Sherlock cluster includes 6 GPU nodes with dual socket Intel(R) Xeon(R) CPU E5–2640 v2 @ 2.00GHz; 256 GB RAM; 200 GB local storage.

### Classification accuracies and confusion matrix

#### Individual and knowledge-based amino acid group accuracy

Prediction accuracies of the models are evaluated using two different metrics: individual class accuracy and knowledge-based group accuracy. Individual class accuracy measures the probability of the network to predict the exact amino acid as the correct class. Since it is known that chemically similar amino acids tend to substitute each other in naturally occurring proteins, to further evaluate the ability of the network to capture known amino acid biochemical similarity, we also calculate a knowledge-based group accuracy metric based on predefined amino acid groupings [41]. For group accuracy, a prediction is considered correct if it is within the knowledge-based amino acid group as the true class.

#### Confusion matrix

Upon the completion of model training, the model weights can then be used to perform prediction for any input local protein box. For a given set of input examples, the number of examples that have true labels i and are predicted as label j is recorded in the position [i, j] of the raw count confusion matrix M. To obtain the probability of examples of true label i being predicted as label j, each row i of the raw count confusion matrix M is then normalized by the total number of examples having the true label i to generate the row-normalized confusion matrix *N*_*row*_, where each number in *N*_*row*_ has a value between 0 ~ 1 and the sum of each row equals 1.

5 $$ {N}_{row}\left[ i, j\right]= M\left[ i, j\right]/{\sum}_j M\left[ i, j\right] $$

The above described process is applied to the training and test dataset to generate 2 separate row-normalized confusion matrices. The matrices are then plot as heat maps using the Matplotlib package.

### Clustering

To identify amino acid environment groups discovered by the network, we performed hierarchical clustering [42] on the row-normalized confusion matrices of both the train and test dataset. Hierarchical clustering with the Ward linkage method was performed using the scipy.cluster.hierarchy package [43].

### Structure-based substitution matrix

Conventional sequence-based substitution matrices such as BLOSUM62 and PAM250 are calculated from the log odd ratio of substitution frequencies among multiple sequence alignments within defined sequence databases. Using an analogous concept, we construct a frequency-based, structure-based substitution matrix from our raw count confusion matrix M. We generated a second matrix considering the score matrix as a measure of similarity between any two amino acid types. This matrix is derived based on dot product similarities between entries of amino acid microenvironment pairs in the raw count confusion matrix. The two score matrices are denoted as *S*_*freq*_ and *S*_*dot*_ respectively, and are calculated using the following equations.

#### Score matrix I: Frequency-based score

The frequency-based substitution scores were calculated using the following equations:

$$ \begin{array}{l} p\left( i, j\right)= M\left[ i, j\right]/{\sum}_i{\sum}_j M\left[ i, j\right]\ \hfill \\ {}{q}_{row}(i)={\sum}_j M\left[ i, j\right]/{\sum}_i{\sum}_j M\left[ i, j\right]\ \hfill \\ {}{q}_{col}(j)={\sum}_i M\left[ i, j\right]/{\sum}_i{\sum}_j M\left[ i, j\right]\hfill \\ {}{S}_{freq\hbox{'}}=\mathit{\log}\left\{ p\left( i, j\right)/{q}_{row}(i)\ast {q}_{col}(j)\right\}\hfill \end{array} $$

To enable straight-forward comparison to other substitution matrices, we create a symmetric substitution matrix by averaging over the original and transposed *S*_*freq*_ as below.

$$ {S}_{freq}=\left({S}_{freq\prime }+{S_{freq\prime}}^T\right)/2 $$

#### Score matrix II: Dot-product-based score

The dot-product based scores were calculated using the following equations

$$ \begin{array}{l}{N}_{row}\left[ i, j\right]= M\left[ i, j\right]/{\sum}_j M\left[ i, j\right]\hfill \\ {}{N}_{col}\left[ i, j\right]= M\left[ i, j\right]/{\sum}_i M\left[ i, j\right]\hfill \\ {}{Row}_i={N}_{row}\left[ i,:\right]/\sqrt{\sum_k{\left({N}_{row}\left[ i, k\right]\right)}^2}\hfill \\ {}{Row}_j={N}_{row}\left[ j,:\right]/\sqrt{\sum_k{\left({N}_{row}\left[ j, k\right]\right)}^2}\hfill \\ {}{Col}_i={N}_{col}\left[:, i\right]/\sqrt{\sum_k{\left({N}_{col}\left[ k, i\right]\right)}^2}\hfill \\ {}{Col}_j={N}_{col}\left[:, j\right]/\sqrt{\sum_k{\left({N}_{col}\left[ k, j\right]\right)}^2}\hfill \\ {}{S}_{dot}\ \left[ i, j\right]=\mathit{\log}\left\{ dot\left({Row}_i,{Row}_j\right)+ dot\left({Col}_i,{Col}_j\right)\right\}\hfill \end{array} $$

The two score matrices are calculated for both the training and test predictions and are denoted as *S*_*freq* − *train*_, *S*_*freq* − *test*_, *S*_*dot* − *train*_, *S*_*dot* − *test*_, respectively. Because similar scores were obtained between the training and the test predictions, *S*_*freq* − *train*_ and *S*_*dot* − *train*_are used are representative matrices and are denoted as *S*_*freq*_ and *S*_*dot*_. Comparison between the matrices to BLOSUM62, and PAM250, and WAC were performed using linear least-square regressions using the scipy.stats.linregress module.

### T4 mutant classification

#### T4 lysozyme mutant and wild type structures

The PDB IDs of 40 T4 lysozyme mutant structures were obtained from the SCOPe2.6 database [44] and the corresponding 3D structures are downloaded from the PDB. We categorize the effects of the mutants based on their associated literature, where a stabilizing mutation is categorized as “neutral” and a destabilizing mutation is categorized as “destabilizing”. Table 2 summarizes the 40 mutant structures employed in this study. To compare between the microenvironments surrounding the wild type and mutated amino acids, the wild type T4 lysozyme structure (PDB ID: 2lzm [45]) is also employed.

**Table 2:** Summary of the 40 T4 mutant structure

Variant	Mutant PDB ID	Effect	Source
G77A	1 L23	Neutral	[64]
A82P	1 L24	Neutral	[64]
A93T	129 L	Neutral	[65]
T151S	130 L	Neutral	[65]
T26S	131 L	Neutral	[65]
V149 M	1CV6	Neutral	[66]
V87 M	1CU3	Destabilizing	[66]
S38 N	1 L61	Neutral	[67]
T109 N	1 L59	Neutral	[67]
T109D	1 L62	Neutral	[67]
N116D	1 L57	Neutral	[67]
D92N	1 L55	Destabilizing	[67]
S38D	1 L19	Neutral	[68]
N144D	1 L20	Neutral	[68]
M106I	1P46	Neutral	[69]
M120Y	1P6Y	Neutral	[69]
V149I	1G0Q	Neutral	[70]
T152 V	1G0L	Neutral	[70]
V149S	1G06	Destabilizing	[70]
V149C	1G07	Destabilizing	[70]
V149G	1G0P	Destabilizing	[70]
E108V	1QUG	Neutral	[71]
L99G	1QUD	Destabilizing	[71]
S117F	1TLA	Neutral	[72]
M106 L	234 L	Neutral	[73]
M120 L	233 L	Neutral	[73]
M106 K	231 L	Destabilizing	[73]
M120 K	232 L	Destabilizing	[73]
I3V	1 L17	Neutral	[74]
I3Y	1 L18	Destabilizing	[74]
M102 K	1 L54	Destabilizing	[75]
T157I	1 L10	Destabilizing	[76]
G156D	1 L16	Destabilizing	[77]
R96H	1 L34	Destabilizing	[78]
I3P	1 L97	Destabilizing	[79]
R96N	3CDT	Destabilizing	[80]
R96D	3C8Q	Destabilizing	[80]
R96W	3FI5	Destabilizing	[80]
R96Y	3C80	Destabilizing	[80]
M102 L	1 L77	Destabilizing	[81]

Forty available T4 lysozyme mutant structures were collected and categorized for their effects. Each mutant is classified based on the literature, where a stabilizing mutation is categorized as “neutral” and a destabilizing mutation is categorized as “destabilizing”

#### T4 wild type and mutant structure microenvironment prediction

For each of the selected 40 T4 lysozyme mutant structures, we extract a local box centered on the Cβ atom of the mutated residue, removing side chain atoms of the mutated residue. The same labeling and featurization procedures described in (A) Atom-Channel Dataset section is applied to the extracted box. Wild type counterparts of these 40 mutated residues can be found by mapping the mutated residue number to the wild type structure. Local boxes surrounding the wild type amino acids can then be similarly extracted and featurized. Each pair of wild type and mutant boxes are then fed into the trained 3DCNN for prediction. The predicted labels for wild type and mutant boxes are denoted as WP (wild type predicted) and MP (mutant predicted), respectively.

#### T4 mutation classifier

We built Lasso [46] and SVM [47] classifiers with 4-fold cross validation using the following three sets of features for five different scoring matrices (BLOSUM62, PAM250, WAC, *S*_*freq*_ and *S*_*dot*_), resulting in fifteen different models.

##### Input Features for the T4 mutation classifiers

$$ \begin{array}{l}6\hbox{-} \mathrm{Feature}=\left[\mathrm{S}\left(\mathrm{WT},\mathrm{WP}\right),\mathrm{S}\left(\mathrm{WT},\mathrm{MT}\right),\mathrm{S}\left(\mathrm{WT},\mathrm{MP}\right),\mathrm{S}\left(\mathrm{WP},\mathrm{MT}\right),\mathrm{S}\left(\mathrm{WP},\mathrm{MP}\right),\mathrm{S}\left(\mathrm{MT},\mathrm{MP}\right)\right]\hfill \\ {}3\hbox{-} \mathrm{Feature}=\left[\mathrm{S}\left(\mathrm{WT},\mathrm{WP}\right),\mathrm{S}\left(\mathrm{WT},\mathrm{MT}\right),\mathrm{S}\left(\mathrm{WP},\mathrm{MT}\right)\right]\hfill \\ {}1\hbox{-} \mathrm{Feature}=\left[\mathrm{S}\left(\mathrm{WT},\mathrm{MT}\right)\right]\hfill \end{array} $$

*S(i,j) is the similarity score taken from the (i,j) element of a score matrix

*WT, WP, MT and MP denote the wild type true label, wild type predicted label, mutant true label, and mutant predicted label, respectively.

The SVM models were constructed using the sklearn.svm package using the Radial Basis Function (RBF) kernel, and the Lasso models were built using the sklearn.linear_model.Lasso package.

### Network visualization: Atom importance map

Our input importance map shows the contribution of each atom to the final classification decision by displaying the importance score of each atom in heat map colors. Importance scores are calculated by first deriving the saliency map described in [48]. Briefly, the saliency map calculates the derivative of the true class score of the example with respect to the input variable I at the point I_0_, where I_0_ denotes the input value. The saliency map is then multiplied by I_0_ to obtain the importance scores for each input voxel for each atom channel. By first order Taylor approximation, the importance score of each atom approximates the effect on the true class score when removing the corresponding atom from the input. Absolute values of the importance scores are recorded, normalized to range (0,100) for each input example across all positions and all channels, and assigned to the corresponding atoms in the local protein box. We visualized results using Pymol [49] by setting the b-factor field of the atoms to the normalized-absolute-valued importance scores. Gradients of the score function with respect to the input variables are calculated by the Theano auto differentiation function.

## Results

### Datasets

Following the procedure described in section T4-lysozyme Free, Protein-Family-Based Training and Test Protein Structure Sets section, we generate a protein structure set that contains 3696 training and 194 test protein families. This results in 32,760 and 1601 training and test structures. Atom-Channel Dataset and FEATURE Dataset are built from the protein structure set to enable comparisons between deep learning based features and conventional hand-engineered features. Atom-Channel Dataset is constructed as described in (A) Atom-Channel Dataset section. The final dataset contains 722,000 training, 38,000 validation and 36,000 test examples, each comprises an approximately equal number of examples from all the 20 amino acid microenvironment types. FEATURE Dataset is constructed as described in (B) FEATURE Dataset section. The resulting datasets are similarly balanced and zero-mean normalized and the final dataset contains 718,200 training, 37,800 validation and 36,000 test examples.

### Network architecture

Our resulting networks are summarized in Table 3. The deep 3D convolutional neural network begins with a 3D convolutional layer, followed by two sequential alternating 3D convolutional and 3D max pooling layers, continued with two fully-connected layers, and ends with a Softmax classifier layer. In this framework, the 3D convolution/max pooling layers, the fully connected layers and the Softmax classifier correspond to the feature extraction, information integration, and classification stage respectively. In the FEATURE Softmax classifier, the feature extraction stage is completed by the FEATURE program in advance. The FEATURE Softmax model similarly continues with two fully-connected layers, and ends with a Softmax classifier layer. To verify that using a Deep Convolutional Architecture provides advantage over using a simple flat neural network with the same input, we also built a Multi-Layer Perceptron with 2 hidden layers. The resulting network architecture is summarized in Additional file 1: Table S1.

**Table 3:** 3DCNN and FEATURE Softmax Classifier Network Architecture

	3DCNN			FEATURE + SOFTMAX		
Stage	Layer	Size	Output Volume	Layer	Size	Output Volume
Feature Extraction Stage	Input		4*20*20*20	Input FEATURE program		480 features
	3D–Conv	3*3*3, 100 Filters	100*18*18*18			
	Dropout (*p* = 0.3)					
	3D–Conv	3*3*3, 200 Filters	200*16*16*16			
	Dropout (*p* = 0.3)					
	3D–Max Pooling	Stride of 2	200*8*8*8			
	3D–Conv	3*3*3, 400 Filters	400*6*6*6			
	Dropout (*p* = 0.3)					
	3D–Max Pooling	Stride of 2	400*3*3*3			
Information Integration Stage	FC Layer	10800*1000 neurons	1000 neurons	FC Layer	480*100 neurons	100 neurons
	Dropout (*p* = 0.3)			Dropout (*p* = 0.3)		
	FC Layer	1000*100 neurons	100 neurons	FC Layer	100*20 neurons	20 neurons
	Dropout (*p* = 0.3)			Dropout (*p* = 0.3)		
Classification Stage	Softmax Classifier	100 neurons*20 classes	20 scores	Softmax Classifier	20 neurons* 20 classes	20 scores

The Stage column describes the component stages for the deep 3DCNN and FEATURE Softmax models. In our 3DCNN, the 3D convolution and max pooling layers, the fully connected layers, and the Softmax classifier correspond to the feature extraction, information integration, and classification stage respectively. In the FEATURE Softmax classifier, the feature extraction stage is completed by the FEATURE program in advance. The Layer column describes the type of layer employed in each stage for each model, where 3D–Conv represents 3D convolutional layer, 3D Max-Pooling represents 3D max pooling operation with stride of 2, Dropout represents dropout operation with *p* = 0.3, and FC Layer stands for fully-connected layer. The Size column further describes the parameters used in each layer. For 3D–Conv layers, the number of filters in each layer and the size of the receptive fields of the filters are specified. For 3D Max-Pooling layers, a stride of 2 is used. For FC Layers, M*N neurons specifies the number of input and output neurons, respectively. The Output volume column describes the size of output of each layer. For 3D–conv and 3D–Max Pool layers, the output is a 4D tensor, where the numbers describe the number of channels, output height, output width, and output depth, respectively. For FC Layer, the output is a vector, and the number describes the number of output neurons

### 20 amino acid classification accuracies and confusion matrix

**Fig. 5:**
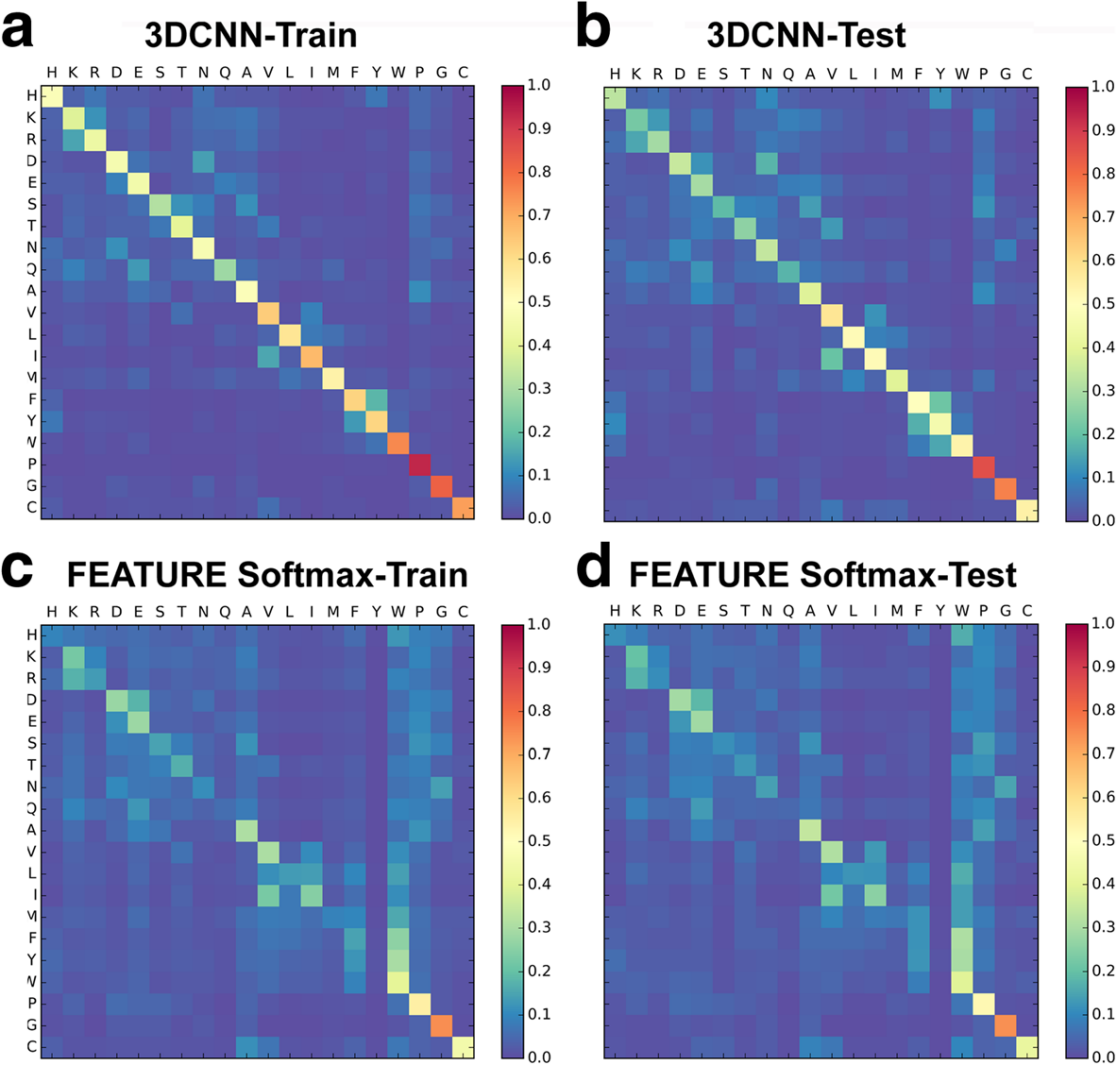
Confusion matrices for predictions of the 20 amino acid microenvironments. Predictions on the training and test datasets using 3DCNN and FEATURE Softmax Classifier are summarized into confusion matrices to inspect the propensity of each microenvironment type to be predicted as one another. The 20 amino acids are arranged according to knowledge-based amino acid groups, where amino acids known to be biochemically similar are adjacent. The ith, jth element of the matrices shows the probability of examples of true label i being predicted as label j. The probability is represented in heat map colors. **a** 3DCNN-Train. **b** 3DCNN-Test. Local block structures in the confusion matrices for 3DCNN demonstrate similarities and differences between amino acid microenvironments. For example, phenylalanine (F), tryptophan (W), and tyrosine (Y) form a hydrophobic and aromatic block. Similar block structure is observed for test predictions. The captured features are robust across protein families. **c** FEATURE-Train. **d** FEATURE-Test. Block structures are less evident in the confusion matrices for the FEATURE Softmax classifier

To classify the 20 amino acid microenvironment, we trained the deep 3DCNN and the MLP on the Atom-Channel dataset and the FEATURE Softmax classifier on the FEATURE Dataset, respectively. Results of the individual and knowledge-based group classification accuracies of 3DCNN and the FEATURE Softmax classifier are reported in Table 4. Comparisons between the performances of 3DCNN and MLP are reported in Additional file 2: Table S2. To inspect the propensity of each microenvironment type to be predicted as the other 19 microenvironment types, Fig. 5 shows heat maps for the confusion matrices generated from predictions on the training and test datasets using the 3DCNN and the FEATURE Softmax classifier, where the ith, jth element of the matrices contains the probability of examples of true label i being predicted as label j.

**Table 4:** Individual and knowledge-based group classification accuracies of 3DCNN and the FEATURE Softmax classifier

Method – Dataset	Single Class accuracy	Knowledge-Based Group Accuracy
3DCNN- train	0.55	0.6732
3DCNN- test	0.425	0.573
FEATURE vectors -train	0.245	0.416
FEATUR vectors -test	0.237	0.405

The deep 3DCNN achieves superior prediction performance compared to models that employ conventional structure-based hand-engineered biochemical features. A two-fold increase in prediction accuracies is achieved by the 3DCNN compared to the FEATURE Softmax Classifier. 3DCNN correctly predict amino acid types for structures in the test dataset, which are in proteins families different from the ones in the training dataset

### Amino acid clustering

**Fig. 6:**
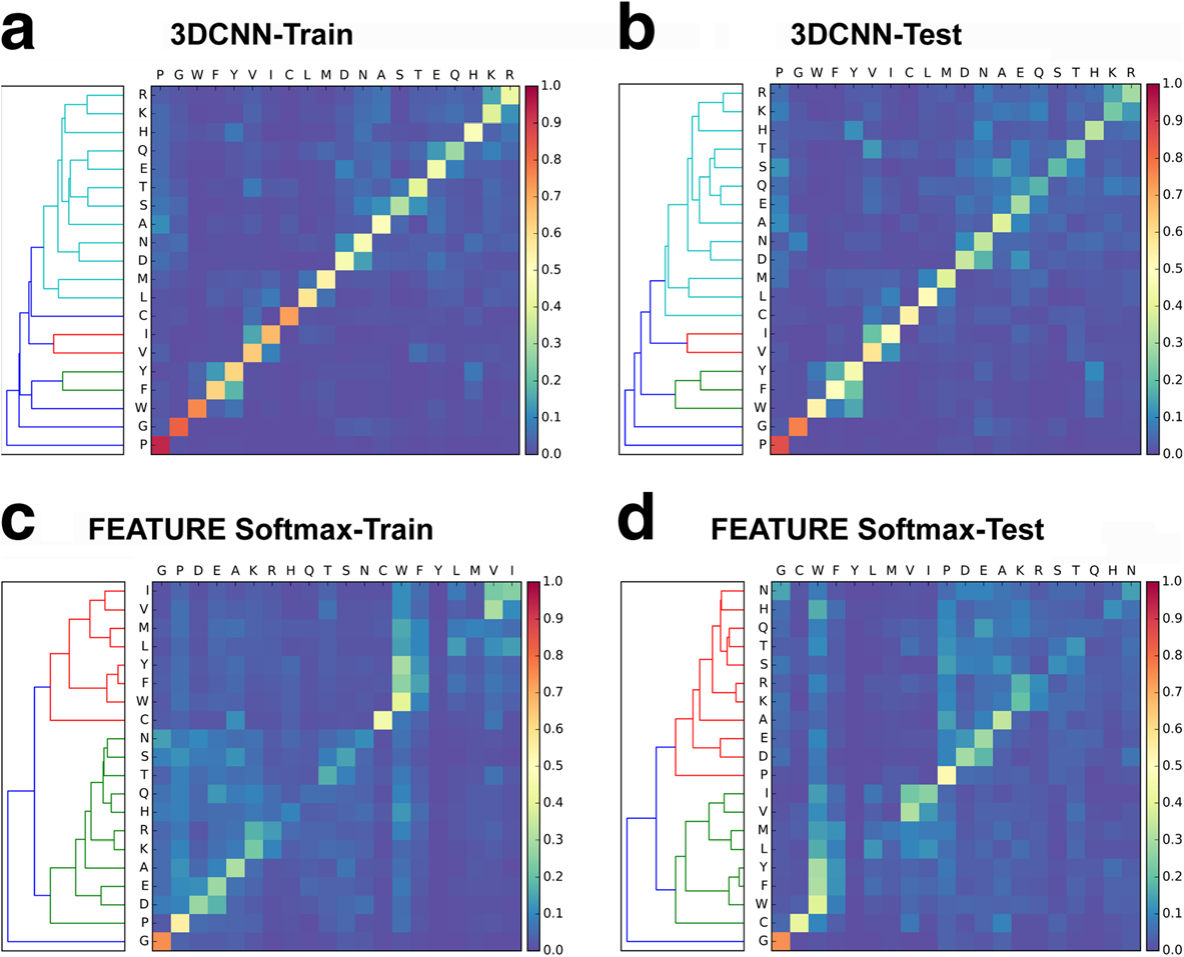
Hierarchical Clustering of normalized confusion matrices. The ith, jth element of the row-normalized matrices shows the probability of examples of true label i being predicted as label j. The probability is represented in heat map colors. Hierarchical clustering reveals similarities between amino acid microenvironments in terms of their propensities to be assigned to the 20 amino acid types. **a** 3DCNN-Training. Amino acid groupings discovered by our 3DCNN generally agree with known amino acid similarities. Six clusters were discovered by our network. The first cluster includes phenylalanine, tryptophan, and tyrosine. These are the three amino acids known to be hydrophobic and aromatic. The second and third clusters comprises valine, isoleucine and leucine, methionine respectively, which are all non-polar and aliphatic. The polar amino acids form the fourth cluster. Amino acids with known distinct properties, glycine and cysteine do not form local blocks with the other amino acids. **b** 3DCNN-Test Groupings generated for the test examples are consistent with the training counterparts. **c** FEATURE-Softmax-Training. **d** FEATURE-Softmax-Test Clustering on the FEATURE Softmax classifier generates much coarser amino acid groupings than the ones discovered by 3DCNN. The two major groups, hydrophobic and polar amino acids are separated

In 20 Amino Acid Classification Accuracies and Confusion Matrix section, we inspected the group prediction accuracy based on knowledge based amino acid groups. To identify amino acid microenvironment groups automatically discovered by the network, hierarchical clustering was performed on the row-normalized confusion matrices. The results are shown in Fig. 6.

### Structure-based substitution matrix

**Fig. 7:**
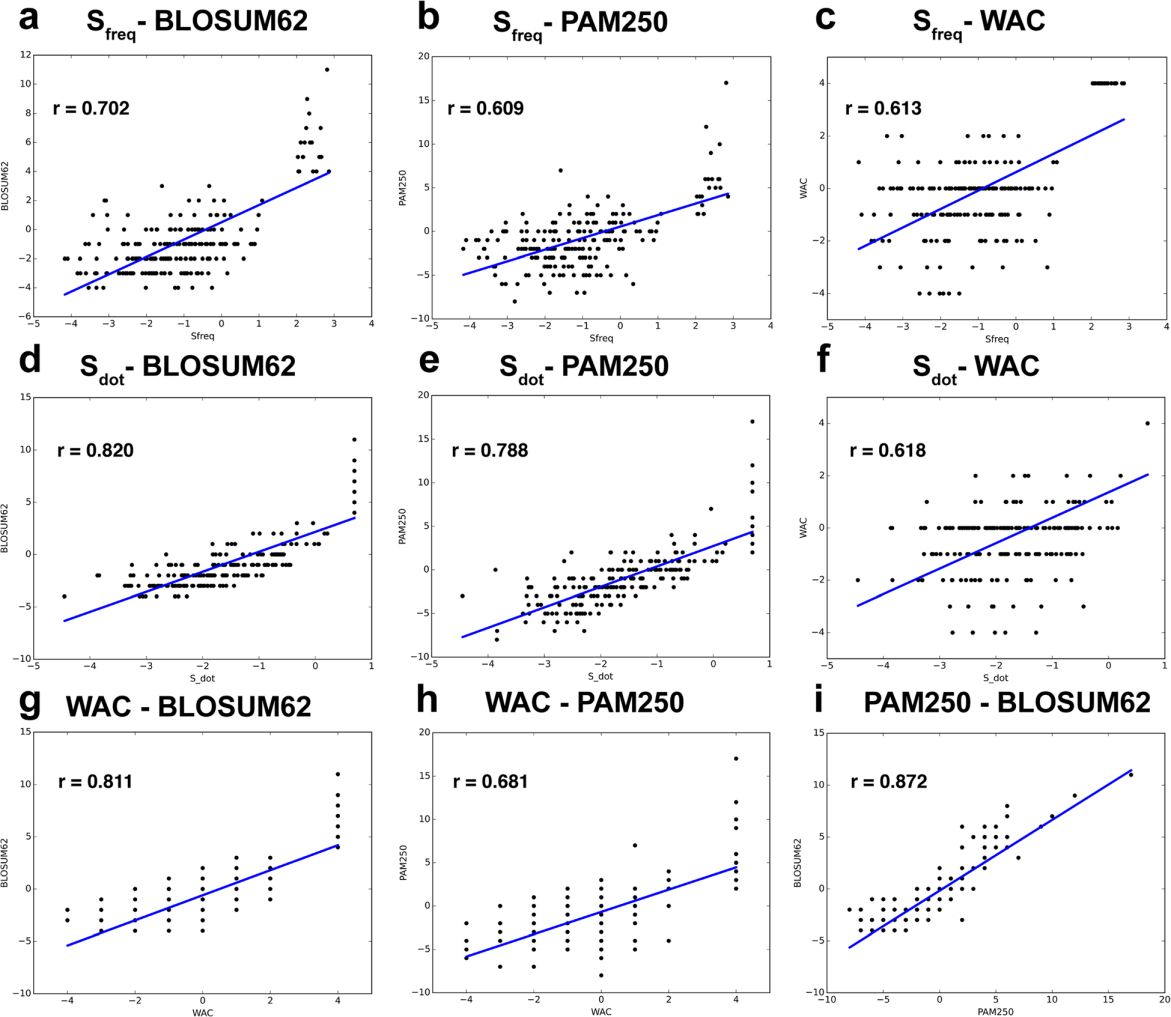
Scatter plots of similarity scores between *S*_*freq*_, *S*_*dot*_ and benchmarking matrices BLOSUM62, PAM250, WAC**. a** *S*_*freq*_- BLOSUM62. **b** *S*_*freq*_- PAM250. **c** *S*_*freq*_- WAC. **d** *S*_*dot*_- BLOSUM62. **e** *S*_*dot*_- PAM250. **f** *S*_*dot*_-WAC. **g** WAC- BLOSUM62. **h** WAC- PAM250. **i** PAM250- BLOSUM62. *S*_*freq*_ shows generally good correlations with BLOSUM62 and PAM250; *S*_*dot*_ shows strong correlation with BLOSUM62 and PAM250; *S*_*freq*_ and *S*_*dot*_ show no significant correlations with WAC

We derived the 3DCNN-frequency-based (*S*_*freq*_) and the 3DCNN-dot-product-based (*S*_*dot*_) substitution matrices from our raw count confusion matrix following the procedure described in Structure-based Substitution Matrix section. Comparison between the two matrices to BLOSUM62, and PAM250, and WAC were performed using linear least-square regressions. We also calculate correlations between BLOSUM62, and PAM250, and WAC for benchmarking purpose. The least square coefficients are summarized in Table 5 and the scatter plots are shown in Fig. 7.

**Table 5:** Correlation between deep learning derived substitution matrices and benchmarking matrices

Matrix 1	Matrix 2	R-value
3DCNN- *S*_*freq*_	BLOSUM62	0.702
3DCNN- *S*_*freq*_	PAM250	0.609
3DCNN- *S*_*freq*_	WAC	0.613
3DCNN- *S*_*dot*_	BLOSUM62	**0.82**
3DCNN- *S*_*dot*_	PAM250	**0.788**
3DCNN- *S*_*dot*_	WAC	0.6183
WAC	BLOSUM62	**0.811**
WAC	PAM250	**0.681**
PAM250	BLOSUM62	**0.872**

We show pairwise comparison of multiple matrices, and report the R-value of the correlation

### T4 mutant classification

Forty available T4 lysozyme mutant structures were collected and categorized for their effects. Each mutant is categorized as either destabilizing or neutral. We use our network to predict the optimal residue type for both the wild type and mutant structures at the corresponding variant sites. Each site can be summarized using their true labels and prediction results in the following form: [wild type true (WT), wild type prediction (WP), mutant true (MT), mutant prediction (MP)]. The results for the 40 sites are summarized in Table 6.

**Table 6:**
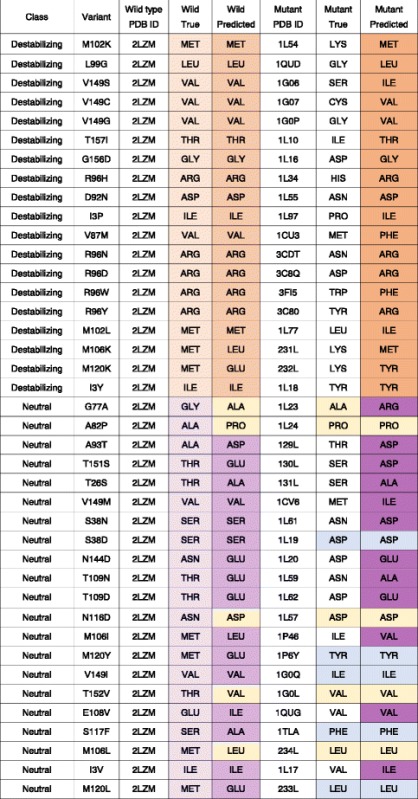
Predicted and true residue type for the wild type and mutant structures at the variant sites

Distinct prediction patterns can be observed between the destabilizing and neutral variants. For the destabilizing variants, our network makes correct predictions with very high confidence on the wild type microenvironments and the predictions on the mutant microenvironments often resemble the true wild type residues, as highlighted in orange. On the other hand, predictions on wild type environment vary significantly. Predictions on mutant microenvironment do not resemble the wild type amino acid, but are rather more similar to the mutant amino acid type, as highlighted in blue. For some cases, predictions of the wild type environment even match exactly to the mutant residues, as highlighted in yellow. These findings suggest that destabilizing mutations happens in microenvironments where the wild type amino acid are strongly preferred while the neutral ones tend to be observed when amino acids other than the wild type are tolerated or even preferred

We subsequently built classifiers to predict whether a mutation has a destabilizing or neutral effect. Specifically, we first used the *S*_*freq*_, *S*_*dot*_, BLOSUM62, PAM250, and the WAC similarity matrices to generate the 6-Feature, 3-Feature, and 1-Feature sets, as described in T4 mutation classifier section. Lasso and SVM classifiers using the 15 sets of features was trained with 4-fold cross validation. The results are summarized in Table 7.

**Table 7:** Prediction accuracies of T4 mutant classifiers

Features	4 fold cross-validation			
	Lasso Train	Lasso Test	SVM Train	SVM Test
6-Feature- *S*_*freq*_	0.90	0.825	0.967	0.825
6-Feature- *S*_*dot*_	0.875	0.85	0.933	0.825
6-Feature- S_BLOSUM_	0.883	0.85	0.967	0.775
6-Feature- S_PAM_	0.883	0.875	0.958	0.775
6-Feature- S_WAC_	0.85	0.825	0.925	0.775
3-Feature- *S*_*freq*_	0.808	0.775	0.833	0.825
3-Feature- *S*_*dot*_	0.800	0.825	0.858	0.775
3-Feature- S_BLOSUM_	0.817	0.800	0.858	0.825
3-Feature- S_PAM_	0.867	0.850	0.917	0.80
3-Feature- S_WAC_	0.825	0.825	0.858	0.825
1-Feature- *S*_*freq*_	0.725	*0.675*	0.724	*0.7*
1-Feature- *S*_*dot*_	0.708	*0.675*	0.742	*0.725*
1-Feature- S_BLOSUM_	0.633	0.575	0.667	0.475
1-Feature- S_PAM_	0.525	0.525	0.708	0.4
1-Feature- S_WAC_	0.525	0.525	0.633	0.5

Performances of Lasso and SVM models built with 1-Feature, 3-Feature, and 6-Feature set from 5 different matrices are compared. The 6-Feature set comprises the substitution scores indexed by the six pairs of true and predicted class for the wild type and mutant variant microenvironment. Specifically, 6-Feature set = [S(WT,WP), S(WT,MT), S(WT,MP), S(WP,MT), S(WP,MP),S(MT,MP)], where S(i,j) is the similarity score taken from the (i,j) element of a score matrix, WT, WP, MT and MP denote the wild type true label, wild type predicted label, mutant true label, and mutant predicted label, respectively. The 3-Feature set is composed of [S(WT,WP), S(WT,MT), S(WP,MT)] and the 1-Feature set only contains [S(WT,MT)]

S_freq_ and S_dot_  matrices show significant advantage with the 1-Feature set (highlighted in boldface), when only the wild type true label and the mutant true labels are known. Models using the 3-Feature and 6- Feature sets achieved better prediction accuracies than using the 1- Feature set alone. Significant boosts of performance using the 3-Feature set over the 1-Feature set are observed for models built with BLOSUM and PAM matrices. The addition information of the predicted label for the wild type structure provides key information that was not captured by sequence derived matrices

### Network visualization

**Fig. 8:**
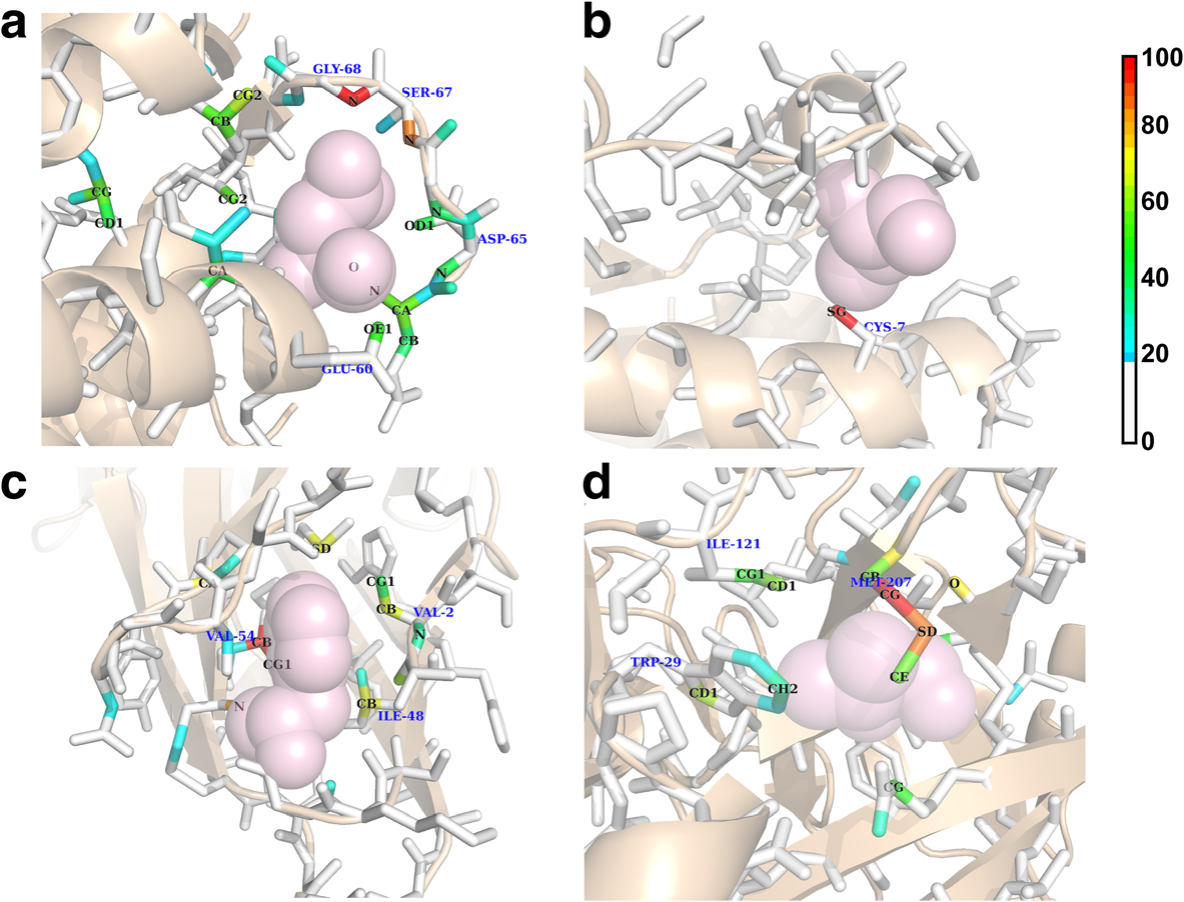
Importance visualization of local amino acid microenvironments. Visualizations of importance scores of each input atom are displayed as heat maps. The color demonstrates how each atom within the local box contribute to the decision. The importance scores range from 0 to 100. Atoms with the lowest important scores (<20) are shown in white, and the red to blue heat map spectrum highlights the most important to the least important atoms. Transparent light pink spheres are drawn to show the space previously occupied by the removed residue. **a** Microenvironment surrounding a key ASP residue at the EF_HAND calcium binding site (PDB: 1A2X, ASP 63.) Our importance score map indicates that the correct prediction relies on the two nitrogen atoms, which are in close proximity to the electronegative oxygen atoms of the removed aspartic acid residue. **b** Microenvironment surrounding a PROSITE INSULIN motif, with the key CYS residue removed (PDB:1IZA, CYS 7.) The 3DCNN network made the correct prediction of CYS primarily based on the SG atom from a nearby cysteine residue. This SG atom originally forms disulfide bond with the SG atom of the removed cysteine residue. The unique disulfide bond pattern was implicitly captured by our network to facilitate the classification. **c** Microenvironment surrounding a phenylalanine residue (PDB: 1ZPL, PHE_52). The three highlighted regions in the heat map are the side-chain atoms of VAL 54, ILE 48, VAL 2, all of which comprise of non-polar carbon atoms. **d** Microenvironment surrounding a valine residue (PDB: 1VJ9 [64], VAL 200). The highlighted atom groups are the side-chains of MET 207, TRP 29 and ILE 121, which are similarly non-polar

To gain insights into what the network has learned, we calculate an importance map to inspect the contribution of each atom to the final classification decision. The importance scores are calculated as described in Network Visualization: Atom Importance Map section. Atoms within the local box are shown as sticks. Visualizations of importance scores of each input atom are displayed as heat maps. Example visualizations are shown in Fig. 8. The color demonstrates how each atom within the local box contributes to the decision. Atoms with the lowest important scores (<20) are shown in white, and the red to blue heat map spectrum highlights the most important to the least important atoms. Transparent light pink spheres are drawn to show the space previously occupied by the removed residue.

## Discussion

### Classification accuracies

The deep 3DCNN achieves superior prediction performance compared to models that employ conventional structure-based hand-engineered biochemical features. As can be seen in Table 4, we achieve a two-fold increase in prediction accuracies using the 3DCNN compared to the FEATURE Softmax Classifier. Importantly, the 3DCNN can correctly predict amino acid types for structures in proteins families that are different from the ones in the training dataset; features learned by the 3DCNN describe fundamental bio-physiochemical properties and are generalizable to all proteins. The significant gap between prediction performances of our 3DCNN with the MLP model reported in Additional file 2: Table S2 shows that with the same training data, the deep 3D convolutional architecture offers advantages over simple flat neural networks.

Among the 20 amino acid microenvironments, our 3DCNN network has the highest prediction accuracies for the C, P and G microenvironments. This is likely due to their distinct and conserved structural properties. Non-polar amino acids tend to have higher prediction accuracies than the polar amino acids by our 3DCNN. Hydrophobic interactions, such as pi-stacking, are often formed within shorter spatial distances than electrostatic interactions between polar amino acids [50, 51]. The bottom-up nature of convolutional layers makes 3DCNNs better at extracting features describing local interactions than the longer-range ones. In this study, we employ a network that uses 3 convolution layers using 3*3*3 Å filters with alternating pooling layers. This creates receptive fields of 12*12*12 Å for each neuron at the final pooling layer. Our predictions therefore depend on combinations of the local features that are at most 12 Å in spatial range within the 20 Å input boxes.

### Confusion matrices and amino acid groupings

Our network captures the similarities and differences between amino acid microenvironments. Figure 5 arranges the 20 amino acids according to knowledge-based amino acid groups, where amino acids known to be biochemically similar are adjacent. Local block structure in the confusion matrices in Fig. 5a and b demonstrates amino acid environment similarities captured by the network. For example, phenylalanine (F), tryptophan (W), and tyrosine (Y) form a hydrophobic and aromatic block. Similar block structures are less evident in the confusion matrices for the FEATURE Softmax classifier although confusion between neighboring amino acids can still be observed.

Hierarchical clustering further demonstrates the extent of similarity captured by our networks. Clustering on the row-normalized confusion matrix reveals similarities between amino acid microenvironments in terms of their propensities to be assigned to the 20 amino acid microenvironment types. Figure 6 shows that the training and test performances are consistent.

Amino acid groupings “discovered” by our 3DCNN generally agree with known amino acid similarities. Hierarchical clustering divides the amino acids into six distinct clusters, as shown in Fig. 6. It is visible in Fig. 6a and b that the polar amino acids histidine (H), lysine (K), arginine (R), aspartic acid (D), glutamic acid (E), serine (S), threonine (T), asparagine (N), glutamine (Q) and a non-polar aliphatic amino acid alanine (A) form a large and weak block, within which K and R; D and N; S and T; and E and Q, form smaller and distinct blocks. Similarly, the non-polar amino acids, F, W, Y, V, I, L, M together form a large weak block, within which the three clusters separate. Amino acids with known distinct properties, glycine (G) and cysteine (C) do not form local blocks with the other amino acids. Clustering on the FEATURE Softmax classifier generates much coarser amino acid groupings. The two major groups, hydrophobic and polar amino acids are separated. However, finer grouping within the two groups are less evident.

Results from clustering also reveal interesting aspects of amino acid similarity learned de novo from structural data. Interestingly, alanine is not grouped with valine, isoleucine, leucine, and methionine, as in many classifications based on hydrophobicity. Instead, serine, threonine, and alanine are substituted for one another frequently by our 3DCNN, likely due to their small sizes [52]. However, size and molecular volume do not seem to dominate the biochemical similarities; cysteine and glycine are well separated from serine, threonine, and alanine despite of their similar sizes. Glutamine, methionine, and lysine are of close molecular weight but do not cluster together while lysine and arginine are different size but are grouped together. Isoleucine, valine and threonine are all Cβ branched and are substantially bulkier near the protein backbone. It is likely that isoleucine is grouped together with valine instead of leucine for this reason. As expected, threonine is considered much more similar to serine than to isoleucine and valine. Thus, size, molecular weight, geometry and biochemical properties all contribute to the groupings.

### Structure-based substitution matrix

The prediction statistics over our millions of training and test examples provide information about the general propensity of an amino acid to be substituted for another. We used our prediction statistics to construct two amino acid substitution matrices *S*_*freq*_ and *S*_*dot*_, and compared them to BLOSUM62 and PAM250 as benchmarks. BLOSUM62 and PAM250 are calculated from the log odd ratio of substitution frequencies among multiple sequence alignments within defined sequence databases and are symmetric. We derived *S*_*freq*_ using an analogous frequency-based concept. However, our matrix is not symmetric: substitutability from amino acid microenvironment i to j is different from substitutability from amino acid microenvironment j to i. The expected frequency of confusion from i to j depends on the fraction of examples with true labels i and the propensity of the network to make predictions of j, instead of the fraction of examples with true labels i and true labels j. As a result, the i, j notation in both the numerator and denominator in our odd-ratio equation is not exchangeable, and the resulting matrix is non-symmetric. To enable straightforward comparison to the benchmark matrices, we created a symmetric substitution matrix by averaging over the original and transposed *S*_*freq*′_. As shown in Table 5 and Fig. 7, *S*_*freq*_ have generally good correlations with BLOSUM62 and PAM250.

We also compare our matrices to WAC, a substitution matrix derived using the FEATURE program [29]. The matrix is similarly constructed from the biochemical, biophysical, and structural features around the 20 amino acids, but from a human-engineered-features perspective. The more statistically similar the FEATURE profiles for two amino acids, the higher the similarity score. We built our *S*_*dot*_ matrix similarly from similarities in prediction profiles between amino acid pairs. *S*_*dot*_ shows strong correlation with BLOSUM62 and PAM250. Interestingly, *S*_*dot*_ show no significant correlation with WAC. This suggests 3DCNN and WAC capture different information.

### T4 mutant classification

The tolerance of proteins to mutation depends on the critical interactions between the lost amino acid and its environment and the ability of the new amino acid to re-establish these interactions. We reasoned that the higher the prediction probability the network assigned to the original amino acid class, the less probable any mutation would be tolerated at the position. Conversely, the higher the similarity score is between the wild type and the mutant amino acid pairs, the more likely the mutation will be accepted. A destabilizing mutation may have very strong preference of the wild type amino acid for the wild type microenvironment and low similarity score between the wild type and mutant amino acid pairs. On the other hand, the microenvironment of a neutral variant site may not have a strong preference to the wild type amino acid and might not show a preference between the wild type and mutant amino acids. We tested these ideas by predicting the effects of mutations on T4 lysozyme. T4 lysozyme was chosen because mutations in T4 lysozyme have been deeply investigated [53, 54], and many mutant structures are available in publicly available databases.

We first used 3DCNN to predict the optimal residue type for both the wild type and mutant structures at the variant sites. Table 6 shows distinct prediction patterns between the destabilizing and neutral variants. For the destabilizing variants, our network makes correct predictions with very high confidence on the wild type microenvironments. Strikingly, instead of predicting amino acid classes similar to the mutated amino acid type, predictions on the mutant microenvironments often resemble the true wild type residues. These variant sites microenvironment likely have special structural features uniquely satisfied by the wild type amino acids, as reflected by the high confidence predictions. After the mutation, even with local structural perturbation to accommodate the mutant amino acid, the network is still able to recognize the microenvironment and predict the original amino acid. On the other hand, for the neutral variants, the reverse to wild type is less strong. Predictions on wild type environment vary significantly. In some cases, our network even predicts the amino acid to be mutated to as the optimal amino acid choice for the wild type microenvironment. Also, predictions on mutant microenvironment do not resemble the wild type amino acid, but are rather more similar to the mutant amino acid type.

These findings are consistent with the idea that destabilizing mutations happen in microenvironments where the wild type amino acid are strongly preferred while the neutral ones tend to be observed when amino acids other than the wild type are tolerated or even preferred. We constructed Lasso and SVM classifiers to quantitatively evaluate the ability of our predictions to separate the destabilizing variants from the neutral ones. The input features were created using similarity scores from the substitution matrices, indexed by the wild type and predicted class labels. Table 7 shows the ability of our models to predict outcomes of the mutation variants. Notably, our *S*_*freq*_ and *S*_*dot*_ matrices show significant advantage with the 1-Feature set, when only the wild type true label and the mutant true label are known. Models built from our substitution matrices on average outperform the ones built from BLOSUM62, PAM250 and WAC by 25.4%. The 1-Feature set only uses the similarity score between the wild type amino acid and the mutant amino acid, and does not rely on the predicted class labels of the wild type and mutant microenvironments. The significant gap of performances between our 3DCNN derived matrices and the other matrices suggests that structural data provide information that could not be derived from sequence substitution frequencies alone. Although the WAC matrix is also structure-based and derived from microenvironment information, the performance was noticeably worse than the deep learning derived matrices.

As expected, models using the 3-Feature and 6- Feature sets achieved better prediction accuracies than using the 1- Feature set alone. Importantly, the predicted labels for the wild type structures provide key information for models employing the BLOSUM and PAM matrices. When our class prediction of the wild type microenvironment is available, performances of the BLOSUM62, PAM250 and WAC models on average increase by 35.8%. This is not surprising since the predicted label for the wild type structure provides direct information about the extent the lost amino acid fits the microenvironments. The more “similar” the prediction is to the lost amino acid, the more probable the lost amino acid might have strong interactions with its environment, and the higher the chance a substitution can be harmful, where the similarity is evaluated by the substitution score between the true and predicted amino acids. For the 3-Feature- *S*_*freq*_ and the 3-Feature- *S*_*dot*_ models, this additional information did not provide as large of a boost as the ones observed for the other matrices. Interestingly, including features from the mutant structures did not provide much improvement over the 3-Feature set, suggesting that the wild type environment may matter most and our models can provide useful information without the mutant structures.

### Network visualization

We present four examples of local amino acid microenvironments, including those of charged, polar, and non-polar amino acids. Figure 8a depicts the local microenvironment surrounding a key aspartic acid residue at the EF_HAND calcium binding site [55, 56] (PDB: 1A2X [57], ASP 63.) Transparent light pink spheres show the space previously occupied by the removed key functional aspartic acid residue. Our network correctly predicts the most suited choice of this microenvironment as aspartic acid. Our importance score map indicates that the decision relies on the two nitrogen atoms, which are in close proximity to the electronegative oxygen atoms of the removed aspartic acid residue. Figure 8b shows a microenvironment surrounding a PROSITE [58] INSULIN motif [59], with the key CYS residue removed (PDB:1IZA [60], CYS 7.) The 3DCNN network correctly predicts cysteine as the most suited amino acid to place in this microenvironment and the decision was made primarily based on the SG atom from a nearby cysteine residue. This SG atom originally forms disulfide bond with the SG atom of the removed cysteine residue. Figure 8c shows an example of a microenvironment surrounding a phenylalanine residue (PDB: 1ZPL [61], PHE 52). Phenylalanine belongs to the non-polar and aromatic group and its six-member ring can form favorable interactions with other non-polar groups. Our network correctly predicts the most suitable residue for the microenvironment as phenylalanine. The three highlighted regions in the heat map are the side-chain atoms of VAL 54, ILE 48, VAL 2, all of which comprise of non-polar carbon atoms. Valine belongs to the non-polar and aliphatic group. Figure 8d shows an example of a microenvironment surrounding a valine residue (PDB: 1VJ9 [62], VAL 200). The highlighted atom groups are the side-chains of MET 207, TRP 29 and ILE 121, which are similarly non-polar.

### Network architecture design

Our network architecture consists of three 3D convolution layers, each using an increasing number of 3*3*3 filters. We did not experiment extensively with different number of 3D convolutional layers and different filter sizes. Small 3*3*3 filters are generally preferable given enough computational power because larger features can be composed from smaller features trough a hierarchical manner. More layers can increase model capacity and the spatial receptive field of each final layer neuron since 3D filters in increasingly higher level layers are looking at an increasingly larger spatial section of the original atomic input space. It would be interesting to see if additional layers or larger filter size can help capitulate longer range electrostatic interactions, as discussed in the Classification Accuracies Section. Max-Pooling layers reduces the dimension of the input and therefore can help reduce the computational expense. More importantly, it reduces the sensitivity of the network to the absolute position of each biochemical feature and therefore increase the translational and rotational invariance of the network. To avoid losing important relative geometry information at the atomic level, the 3D Max Pooling operation was not used immediately after the first 3D Convolutional Layer. We delay the employment of the 3D Max-Pooling layers till the second 3D Convolutional stage where the features are more abstract and less dependent on the absolute spatial orientation.

Comparing our deep 3DCNN to a simple flat neural network, our results show that the convolution architecture offers advantages. The convolutional architecture enforces the local filters to share weights across different locations in the input space, therefore significantly reducing the number of trainable parameters. The local filters enforce the features to be comprised of local spatial features that are recurrently observed and are important for classification instead of allowing the model to memorize combinations of input. These together reduce the tendency of over-fitting for our 3DCNN and allow better performance.

### Input Featurization

Two additional considerations for our 3DCNN performance are the dimension of the local box and input representation. The local box size defines the information accessible by the network and therefore is a hyper-parameter in our framework. Here we extract local protein boxes of 20 Å based on our previous experience with the FEATURE program. FEATURE uses a sphere of diameter of 16 Å to define local microenvironment around functional atoms of each of the 20 amino acid because beyond a 16–20 Å cutoff, the atomic details do not provide significant additional information. We centered our box on Cβ regardless of the amino acid type and enlarged our box by 4 Å to include equivalent surrounding information. For input representation, we divide boxes into grid voxels in this study. The biggest limitation of this design choice is that the grid voxel system is not rotationally invariant. Therefore, we aligned all local boxes in a standard manner using backbone atoms of the central residue to ensure similar orientation. The fine-grain feature extraction procedure enabled our network to achieve good performance in characterizing the amino acid environment.

## Conclusion

To our knowledge, this is the first paper that performs head-to-head comparisons between models utilizing hand-engineered features and end-to-end trained deep learning networks in the context of protein engineering. The consistent success of our deep 3DCNN over methods using human-engineered features suggests that the freedom to discover arbitrary features from raw data provides advantages over pre-defined features. Our results suggest that 3DCNN framework is well suited for analysis of protein microenvironments, and that many of the benefits of CNNs for 2D image analysis accrue in the context of 3D protein analysis. The deep learning framework may hold promise for more advanced protein analyses such as pocket similarity evaluation or predicting protein-protein interactions as more structural data become available.

## Additional files

Additional file 1: Table S1. 3DCNN and MLP Network Architecture. Table summarizing the network architectures of 3DCNN and MLP. (DOCX 15 kb)

Additional file 2: Table S2. Individual and knowledge-based group classification accuracies of 3DCNN and MLP. Summary of the individual and knowledge-based group classification accuracies of 3DCNN and MLP. The deep 3DCNN achieves superior prediction performance compared to the MLP model, demonstrating the advantage of the deep 3D convolutional architecture over a simple flat neural network with the same input. (DOCX 12 kb)

## Abbreviations

3DCNN: Three dimensional convolutional neural network
A: Alanine
BLOSUM: BLOcks SUbstitution Matrix
C, CYS: Cysteine
CNN: Convolutional neural network
D: Aspartate
E: Glutamate
F, PHE: Phenylalanine
G: Glycine
GPU: Graphics processing unit
H: Histidine
I, ILE: Isoleucine
K: Lysine
L: Leucine
M, MET: Methionine
MLP: Multi-Layer Perceptron
N: Asparagine
P: Proline
PAM: Point Accepted Mutation substitution matrices
PDB: Protein Data Bank
Q: Glutamine
R: Arginine
ReLU: Rectified Linear Unit
S: Serine
SVM: Support vector machine
T: Threonine
V, VAL: Valine
W, TRP: Tryptophan
Y: Tyrosine

## References

[1] N. M. Antikainen and S. F. Martin, “Altering protein specificity: techniques and applications” 10.1016/j.bmc.2005.01.059 15781382

[2] Lefèvre F, Rémy MH, Masson JM (1997). Alanine-stretch scanning mutagenesis: a simple and efficient method to probe protein structure and function. Nucleic Acids res.

[3] Thorn KS, Bogan AA (2001). ASEdb: a database of alanine mutations and their effects on the free energy of binding in protein interactions. Bioinformatics.

[4] Brachman RJ, Levesque HJ (1985). Readings in knowledge representation.

[5] Bengio Y, Courville A, Vincent P (2013). Representation learning: a review and new perspectives. Pattern Anal Mach Intell IEEE Trans.

[6] Bagley SC, Altman RB (1995). Characterizing the microenvironment surrounding protein sites. Protein Sci.

[7] Neshich G (2005). STING report: convenient web-based application for graphic and tabular presentations of protein sequence, structure and function descriptors from the STING database. Nucleic Acids res.

[8] Block P, Paern J, Hüllermeier E, Sanschagrin P, Sotriffer CA, Klebe G (2006). Physicochemical descriptors to discriminate protein-protein interactions in permanent and transient complexes selected by means of machine learning algorithms. Proteins Struct Funct Genet.

[9] Zvelebil MJJM, Sternberg MJE (1988). Analysis and prediction of the location of catalytic residues in enzymes. Protein Eng Des Sel.

[10] Buturovic L, Wong M, Tang GW, Altman RB, Petkovic D (2014). High precision prediction of functional sites in protein structures. PLoS One.

[11] Liu T, Altman RB (2011). Using multiple microenvironments to find similar ligand-binding sites: application to kinase inhibitor binding. PLoS Comput Biol.

[12] Tang GW, Altman RB (2014). Knowledge-based fragment binding prediction. PLoS Comput Biol.

[13] Liang MP, Brutlag DL and Altman RB. “Automated construction of structural motifs for predicting functional sites on protein structures.” Pac Symp Biocomput. 2003; pp. 204–15 10.1142/9789812776303_0020 12603029

[14] Bishop CM (2006). Pattern recognition.

[15] Grabowski M, Chruszcz M, Zimmerman MD, Kirillova O, Minor W (2009). Benefits of structural genomics for drug discovery research. Infect Disord Drug Targets.

[16] LeCun Y, Bengio Y, Hinton G (2015). Deep learning. Nature.

[17] Krizhevsky A, Sutskever I and Hinton GE. “ImageNet classification with deep convolutional neural networks.” Adv Neural Inf Process Syst. 2012; pp. 1–9

[18] Szegedy C. et al., “Going deeper with convolutions,” in Proceedings of the IEEE Computer Society Conference on Computer Vision and Pattern Recognition, 2015, vol. 07–12–June, pp. 1–9

[19] Bahdana D, Bahdanau D, Cho K and Bengio Y. “Neural Machine Translation By Jointly Learning To Align and Translate.” Int Conf Learn Represent. 2015, no. http://arxiv.org/abs/1409.0473, pp. 1–15, 2015 Submitted on 1 Sep 2014 (v1), last revised 19 May 2016 (this version, v7))

[20] Kearnes S, McCloskey K, Berndl M, Pande V, Riley P (2016). Molecular graph convolutions: moving beyond fingerprints. J Comput Aided Mol des.

[21] Duvenaud D (2015). Convolutional networks on graphs for learning molecular fingerprints. Adv Neural Inf Process Syst.

[22] Alipanahi B, Delong A, Weirauch MT, Frey BJ (2015). Predicting the sequence specificities of DNA- and RNA-binding proteins by deep learning. Nat Biotechnol.

[23] Zhou J, Troyanskaya OG (2015). Predicting effects of noncoding variants with deep learning-based sequence model. Nat Methods.

[24] R. Miotto, L. Li, B. A. Kidd, and J. T. Dudley, “Deep patient: an unsupervised representation to predict the future of patients from the electronic health records.,” Sci rep*.*, vol. 6, no. April, p. 26094, 2016. 10.1038/srep26094 PMC4869115 27185194

[25] Le Cun Y (1989). “Handwritten digit recognition with a back-propagation network.” Proceedings of the 2nd International Conference on Neural Information Processing Systems.

[26] Wallach I, Dzamba M, and Heifets A. “AtomNet: A Deep Convolutional Neural Network for Bioactivity Prediction in Structure-based Drug Discover.” arXiv Prepr. arXiv1510.02855. 2015 pp. 1–11

[27] Henikoff S, Henikoff JG (1992). Amino acid substitution matrices from protein blocks. Proceedings of the National Academy of Sciences.

[28] B. C. Dayhoff, M.O., Schwartz, R. and Orcutt, “A Model of Evolutionary Change in Proteins,” in Atlas of protein sequence and structure, Volume 5,., National Biomedical Research Foundation Silver Spring, MD, 1978, pp. 345–358

[29] Wei L, AltmanRB, and Chang JT. “Using the radial distributions of physical features to compare amino acid environments and align amino acid sequences.” Pac Symp Biocomput. 1997; pp. 465–76 9390315

[30] Murzin AG, Brenner SE, Hubbard T, Chothia C (1995). SCOP: a structural classification of proteins database for the investigation of sequences and structures. J Mol Biol.

[31] Brenner SE, Koehl P, Levitt M (2000). The ASTRAL compendium for protein structure and sequence analysis. Nucleic Acids res.

[32] Huang Y, Niu B, Gao Y, Fu L, Li W (2010). CD-HIT suite: a web server for clustering and comparing biological sequences. Bioinformatics.

[33] Bateman A (2015). UniProt: a hub for protein information. Nucleic Acids res.

[34] Scherer D, Müller A, and Behnke S. “Evaluation of pooling operations in convolutional architectures for object recognition.” International Conference on Artificial Neural Networks. Springer Berlin Heidelberg. 2010; LNCS, vol. 6354: pp 92-101

[35] Bridle JS (1990). Probabilistic interpretation of feedforward classification network outputs, with relationships to statistical pattern recognition. Neurocomputing.

[36] Theano Development Team. “Theano: A Python framework for fast computation of mathematical expressions.” arXiv e-prints arXiv.abs/1605.02688. 2016; p. 19

[37] Glorot X, Bordes A, Bengio Y (2011). Deep sparse rectifier neural networks. AISTATS ‘11 Proc 14th Int Conf Artif Intell Stat.

[38] Bottou L. “Large-Scale Machine Learning with Stochastic Gradient Descent.” Proc. COMPSTAT’2010. 2010; pp. 177–186

[39] Rumelhart DE, Hinton GE, Williams RJ (1986). Learning representations by back-propagating errors. Nature.

[40] Srivastava N, Hinton G, Krizhevsky A, Sutskever I, Salakhutdinov R (2014). Dropout: a simple way to prevent neural networks from Overfitting. J Mach Learn res.

[41] Gu J and Bourne PE. Structural bioinformatics. Wiley-Blackwell, Hoboken, New Jersey; 2009.

[42] J. H. Ward, “Hierarchical grouping to optimize an objective function,” J am Stat Assoc, vol. 58, no. 301. pp. 236–244, Mar-1963.

[43] Oliphant TE (2007). Python for scientific computing. Comput Sci Eng.

[44] Chandonia J-M, Fox NK, Brenner SE (2016). SCOPe: Manual Curation and Artifact Removal in the Structural Classification of Proteins – extended Database. J. Mol. Biol.

[45] Weaver LH, Matthews BW (1987). Structure of bacteriophage T4 lysozyme refined at 1.7 Å Resolution. J Mol Biol.

[46] Tibshirani R (1996). Regression selection and shrinkage via the Lasso. J R Stat Soc B.

[47] Cortes C, Vapnik V (1995). Support vector networks. Mach Learn.

[48] Simonyan K, Vedaldi A, and Zisserman A. “Deep Inside Convolutional Networks: Visualising Image Classification Models and Saliency Maps.” Proc Int Conf Learn Represent. 2014

[49] “The PyMOL Molecular Graphics System, Version 1.8 Schrödinger, LLC”

[50] Janiak C (2000). A critical account on π–π stacking in metal complexes with aromatic nitrogen-containing ligands †. Dalton Trans.

[51] Alvarez S (2013). A cartography of the van der Waals territories. Dalt Trans.

[52] Betts MJ, Russell RB. Amino acid properties and consequences of substitutions. In: Bioinformatics for geneticists. Chichester: John Wiley & Sons, Ltd. p. 289–316.

[53] W. A. Baase, L. Liu, D. E. Tronrud, and B. W. Matthews, “Lessons from the lysozyme of phage T4,” Protein Sci, vol. 19, no. 4. Wiley-Blackwell, pp. 631–641, Apr-2010. 10.1002/pro.344 PMC2867005 20095051

[54] Rennell D, Bouvier SE, Hardy LW, Poteete AR (1991). Systematic mutation of bacteriophage T4 lysozyme. J Mol Biol.

[55] Kawasaki H, Kretsinger RH (1995). Calcium-binding proteins 1: EF-hands. Protein Profile.

[56] Moncrief ND, Kretsinger RH, Goodman M (1990). Evolution of EF-hand calcium-modulated proteins. I. Relationships based on amino acid sequences. J Mol Evol.

[57] Vassylyev DG, Takeda S, Wakatsuki S, Maeda K, Maéda Y (1998). Crystal structure of troponin C in complex with troponin I fragment at 2.3-a resolution. Proc Natl Acad Sci U S a.

[58] Sigrist CJA (2010). PROSITE, a protein domain database for functional characterization and annotation. Nucleic Acids res.

[59] Blundell TL, Humbel RE (1980). Hormone families: pancreatic hormones and homologous growth factors. Nature.

[60] Bentley GA (1992). Role of B13 Glu in insulin assembly: the hexamer structure of recombinant mutant (B13 Glu → Gln) insulin. J Mol Biol.

[61] Buts L (2005). Impact of natural variation in bacterial F17G adhesins on crystallization behaviour. Acta Crystallogr Sect D Biol Crystallogr.

[62] Schweinitz A (2004). Design of Novel and Selective Inhibitors of Urokinase-type Plasminogen activator with improved pharmacokinetic properties for use as Antimetastatic agents. J Biol Chem.

[63] I. Halperin, D. S. Glazer, S. Wu, and R. B. Altman, “The FEATURE framework for protein function annotation: modeling new functions, improving performance, and extending to novel applications.,” BMC Genomics, vol. 9 Suppl 2, no. Suppl 2, p. S2, 2008. 10.1186/1471-2164-9-S2-S2 PMC2559884 18831785

[64] B. W. Matthews, H. Nicholson, and W. J. Becktel, “Enhanced protein Thermostability from site-directed mutations that decrease the entropy of unfolding.,” Proc Natl Acad Sci U S a*.*, vol. 84, no. October, pp. 6663–6667, 1987. 10.1073/pnas.84.19.6663 PMC299143 3477797

[65] Pjura P, Matthews BW (1993). Structures of randomly generated mutants of T4 lysozyme show that protein stability can be enhanced by relaxation of strain and by improved hydrogen bonding via bound solvent. Protein Sci.

[66] Gassner NC, Baase WA, Lindstrom JD, Lu J, Dahlquist FW, Matthews BW (1999). Methionine and alanine substitutions show that the formation of wild- type-like structure in the carboxy-terminal domain of T4 lysozyme is a rate- limiting step in folding. Biochemistry.

[67] Nicholson H, Anderson DE, Dao Pin S, Matthews BW (1991). Analysis of the interaction between charged side chains and the .Alpha.-helix dipole using designed thermostable mutants of phage T4 lysozyme. Biochemistry.

[68] Nicholson H, Becktel WJ, Matthews BW (1988). Enhanced protein thermostability from designed mutations that interact with α-helix dipoles. Nature.

[69] Mooers BHM, Datta D, Baase WA, Zollars ES, Mayo SL, Matthews BW (2003). Repacking the Core of T4 lysozyme by automated design. J Mol Biol.

[70] Xu J, Baase WA, Quillin ML, Baldwin EP, Matthews BW (2001). Structural and thermodynamic analysis of the binding of solvent at internal sites in T4 lysozyme. Protein Sci.

[71] Wray JW, Baase WA, Lindstrom JD, Weaver LH, Poteete AR, Matthews BW (1999). Structural analysis of a non-contiguous second-site revertant in T4 lysozyme shows that increasing the rigidity of a protein can enhance its stability. J Mol Biol.

[72] Anderson DE, Hurley JH, Nicholson H, Baase WA, Matthews BW (1993). Hydrophobic core repacking and aromatic-aromatic interaction in the thermostable mutant of T4 lysozyme ser 117 → phe. Protein Sci.

[73] Lipscomb LA (1998). Context-dependent protein stabilization by methionine-to-leucine substitution shown in T4 lysozyme. Protein Sci.

[74] Matsumura M, Becktel WJ, Matthews BW (1988). Hydrophobic stabilization in T4 lysozyme determined directly by multiple substitutions of Ile 3. Nature.

[75] Dao-Pin S, Anderson DE, Baase WA, Dahlquist FW, Matthews BW (1991). Structural and thermodynamic consequences of burying a charged residue within the hydrophobic core of T4 lysozyme. Biochemistry.

[76] Grütter MG, Gray TM, Weaver LH, Alber T, Wilson K, Matthews BW (1987). Structural studies of mutants of the lysozyme of bacteriophage T4: the temperature-sensitive mutant protein Thr157 → Ile. J Mol Biol.

[77] Gray TM, Matthews BW (1987). Structural analysis of the temperature-sensitive mutant of bacteriophage T4 lysozyme, glycine 156----aspartic acid. J Biol Chem.

[78] Weaver LH (1989). High-resolution structure of the temperature-sensitive mutant of phage lysozyme, Arg 96 .Fwdarw. His. Biochemistry.

[79] Dixon MM, Nicholson H, Shewchuk L, Baase WA, Matthews BW (1992). Structure of a hinge-bending bacteriophage T4 lysozyme mutant, Ile3 → pro. J Mol Biol.

[80] Mooers BHM, Baase WA, Wray JW, Matthews BW (2009). Contributions of all 20 amino acids at site 96 to the stability and structure of T4 lysozyme. Protein Sci.

[81] Hurley JH, Baase WA, Matthews BW (1992). Design and structural analysis of alternative hydrophobic core packing arrangements in bacteriophage T4 lysozyme. J Mol Biol.

[82] Berman HM (2000). The protein data bank. Nucleic Acids res.

