# Supplementary material for: 3D deep convolutional neural networks for amino acid environment similarity analysis
Source: BMC Bioinformatics. 2017 Jun 14;18:302. doi: 10.1186/s12859-017-1702-0 (PMC5472009; doi:10.1186/s12859-017-1702-0)
Supplement: Supplementary file 1 — 3DCNN and MLP Network Architecture. Table summarizing the network architectures of 3DCNN and MLP. (DOCX 15 kb) [file 12859_2017_1702_MOESM1_ESM.docx]

**Additional file**

**Table S1. 3DCNN and MLP Network Architecture.**

|  | **3DCNN** | | | **MLP** | | |
| --- | --- | --- | --- | --- | --- | --- |
| **Stage** | **Layer** | **Size** | **Output Volume** | **Layer** | **Size** | **Output Volume** |
| Feature Extraction Stage | Input |  | 4*20*20*20 | Input |  | 4*20*20*20 |
|  |  |  |  | Flatten |  | 32,000 |
|  | 3D-Conv-100-3 | 3*3*3,  100 Filters | 100*18*18*18 |  |  |  |
|  | Dropout  (p = 0.3) |  |  |  |  |  |
|  | 3D-Conv | 3*3*3,  200 Filters | 200*16*16*16 |  |  |  |
|  | Dropout  (p = 0.3) |  |  |  |  |  |
|  | 3D-Max Pooling | Stride of 2 | 200*8*8*8 |  |  |  |
|  | 3D-Conv | 3*3*3,  400 Filters | 400*6*6*6 |  |  |  |
|  | Dropout  (p = 0.3) |  |  |  |  |  |
|  | 3D-Max Pooling | Stride of 2 | 400*3*3*3 |  |  |  |
| Information Integration Stage | FC Layer | 10,800*  1,000 neurons | 1,000 neurons | FC Layer | 32,000* 10,000  neurons | 10,000 neurons |
|  | Dropout  (p = 0.3) |  |  | Dropout  (p = 0.3) |  |  |
|  | FC Layer | 1,000*100 neurons | 100 neurons | FC Layer | 10,000 *100 neurons | 100 neurons |
|  | Dropout  (p = 0.3) |  |  | Dropout  (p = 0.3) |  |  |
| Classification Stage | Softmax Classifier | 100 neurons*  20 classes | 20 scores | Softmax Classifier | 100 neurons* 20 classes | 20 scores |

The Stage column describes the component stages for the deep 3DCNN and MLP models. In our 3DCNN, the 3D convolution and max pooling layers, the fully connected layers, and the Softmax classifier correspond to the feature extraction, information integration, and classification stage respectively. In the MLP, the feature extraction stage is simply a flatten operation. The Layer column describes the type of layer employed in each stage for each model, where 3D-Conv represents 3D convolutional layer, 3D Max-Pooling represents 3D max pooling operation with stride of 2, Dropout represents dropout operation with p=0.3, and FC Layer stands for fully-connected layer. The Size column further describes the parameters used in each layer. For 3D-Conv layers, the number of filters in each layer and the size of the receptive fields of the filters are specified. For 3D Max-Pooling layers, a stride of 2 is used. For FC Layers, M*N neurons specifies the number of input and output neurons, respectively. The Output volume column describes the size of output of each layer. For 3D-conv and 3D-Max Pool layers, the output is a 4D tensor, where the numbers describe the number of channels, output height, output width, and output depth, respectively. For FC Layer, the output is a vector, and the number describes the number of output neurons.
