# Supplementary material for: 3D deep convolutional neural networks for amino acid environment similarity analysis
Source: BMC Bioinformatics. 2017 Jun 14;18:302. doi: 10.1186/s12859-017-1702-0 (PMC5472009; doi:10.1186/s12859-017-1702-0)
Supplement: Supplementary file 2 — Individual and knowledge-based group classification accuracies of 3DCNN and MLP. Summary of the individual and knowledge-based group classification accuracies of 3DCNN and MLP. The deep 3DCNN achieves superior prediction performance compared to the MLP model, demonstrating the advantage of the deep 3D convolutional architecture over a simple flat neural network with the same input. (DOCX 12 kb) [file 12859_2017_1702_MOESM2_ESM.docx]

**Table S2. Individual and knowledge-based group classification accuracies of 3DCNN and MLP.**

| Method | Single Class Accuracy | Knowledge-Based Group Accuracy |
| --- | --- | --- |
| 3DCNN- test accuracy | **0.425** | **0.573** |
| MLP- test accuracy | 0.253 | 0.432 |

The deep 3DCNN achieves superior prediction performance compared to the MLP model. A 0.17 increase in single class prediction accuracies is achieved by the 3DCNN compared to the MLP model, demonstrating the advantage of the deep 3D convolutional architecture over a simple flat neural network with the same input.
